# Supplementary material for: IFNγ causes mitochondrial dysfunction and oxidative stress in myositis
Source: Nat Commun. 2024 Jun 26;15:5403. doi: 10.1038/s41467-024-49460-1 (PMC11208592; doi:10.1038/s41467-024-49460-1)
Supplement: Supplementary file 1 — Supplementary Information [file 41467_2024_49460_MOESM1_ESM.pdf]

Supplementary information for

IFNγ causes mitochondrial dysfunction and oxidative stress in myositis

Authors

Catalina Abad[1], Iago Pinal-Fernandez[2,3], Clement Guillou[4], Gwladys Bourdenet[1], Laurent Drouot[1], Pascal Cosette[4,5], Margherita Giannini[6,7], Lea Debrut[6], Laetitia Jean[1], Sophie Bernard[8], Damien Genty[9], Rachid Zoubairi[1], Isabelle Remy-Jouet[10], Bernard Geny[6,7], Christian Boitard[11], Andrew Mammen[2,3,12], Alain Meyer[6, 7], and Olivier Boyer[1,13]

Affiliations

[1]Univ Rouen Normandie, Inserm, UMR1234, FOCIS Center of Excellence PAn'THER, F-76000, Rouen, France

[2] Muscle Disease Unit, National Institute of Arthritis and Musculoskeletal and Skin Diseases, National Institutes of Health, Bethesda, Maryland, USA

[3]Department of Neurology, Johns Hopkins University School of Medicine, Baltimore, Maryland, USA.

[4]Univ Rouen Normandy, Inserm US 51, CNRS UAR 2026, HeRacLeS PISSARO, F-76000 Rouen, France

[5]Univ Rouen Normandie, INSA Rouen Normandie, CNRS, Normandie Univ, PBS UMR 6270, F-76000 Rouen, France

[6]Translational Medicine Federation of Strasbourg, Team 3072, Faculty of Medicine, University of Strasbourg, Strasbourg, France.

[7]Unité exploration fonctionnelle musculaire-service de physiologie, Centre National de Référence des Maladies Auto-Immunes Systémiques Rares de l'Est et du Sud-Ouest - Service de rhumatologie, Hôpitaux Universitaires de Strasbourg, Strasbourg, France.

[8] Univ Rouen Normandie, Inserm US 51, CNRS UAR 2026, HeRacLeS PRIMACEN US 51 UAR 2026, F-76000 Rouen, France

[9]CHU Rouen, Department of Pathology, F-76000, Rouen, France.

[10]Univ Rouen Normandie, Inserm, UMR1096, BOSS facility, F-76000, Rouen, France.

[11]Cochin Institute, Paris Descartes University, Sorbonne Paris Cité, Inserm U1016, Paris, France.

[12]Department of Medicine, Division of Rheumatology, Johns Hopkins University School of Medicine, Baltimore, Maryland, USA.

[13]CHU Rouen, Department of Immunology and Biotherapy, F-76000, Rouen, France.

Supplementary Data inventory

Supplementary Figure 1. Immunofluorescence labelling of immune cell populations infiltrating muscle sections of *Icos*$^{+/+}$ NOD and *Icos*$^{-/-}$ NOD of 8, 25 and 35 weeks of age.

Supplementary Figure 2. Proteome analysis of muscles from *Icos*$^{+/+}$ NOD vs *Icos*$^{-/-}$ NOD mice of 8, 25 and 35 weeks of age additional data.

Supplementary Figure 3. mRNA expression of genes belonging to the category 'muscle contraction' in *Icos*$^{+/+}$ NOD and *Icos*$^{-/-}$ NOD PROX and ADJ.

Supplementary Figure 4. Spatial transcriptome analysis of *Icos*$^{+/+}$ NOD vs. *Icos*$^{-/-}$ NOD ADJ myofibers.

Supplementary Figure 5. Mitochondrial abundance/stability protein levels from proteome analysis.

Supplementary Figure 6. Anti-IFNγ treatment ameliorates *Icos*$^{-/-}$ NOD mice myositis reducing oxidative stress and inflammation.

Supplementary Figure 7. Description of Catwalk parameters.

Supplementary Figure 8. Immunofluorescence labelling of immune cell populations infiltrating muscle sections of *Icos*$^{-/-}$ NOD upon treatment with anti-IFNγ antibodies.

Supplementary Figure 9. Fibrosis analysis in *Icos*$^{-/-}$ NOD mice.

Supplementary Figure 10. Immunofluorescence labelling of immune cell populations infiltrating muscle sections of *Icos*$^{-/-}$ NOD upon treated with NAC in preventive setting.

Supplementary Figure 11. Curative NAC treatment hampers *Icos*$^{-/-}$ NOD mice myositis progression.

Supplementary Figure 12. Immunofluorescence labelling of immune cell populations infiltrating muscle sections of *Icos*$^{-/-}$ NOD upon treated with NAC in curative setting.

Supplementary Figure 13. NAC treatment improves *Icos*$^{-/-}$ NOD mice mitochondrial functional and morphological features.

Supplementary Figure 14. Correlation of GBP and mitochondrial OXPHOS gene expression in human DM.

Supplementary Figure 15. Expression of mitochondrial genes by myositis patient serotype.

Supplementary Figure 16. Expression of mitochondrial genes by myositis patient sex.

Supplementary Figure 17. Analysis of proteome and transcriptome data focusing on peroxisome.

Supplementary Table 1. Seropositivity for anti-TNNT3 antibodies in *Icos*$^{-/-}$ NOD mice.

Supplementary Table 2. Supplementary information of NT vs DM mitochondrial gene expression analysis.

Supplementary Table 3. Supplementary information of NT vs DM mitochondrial gene expression analysis.

Supplementary Table 4. Serum glucose levels in mice from our treatment studies.

Supplementary Table 5. Sequences of the primers used for qRT-PCR analyses.

Supplementary Table 6. Antibodies used in immunofluorescence.

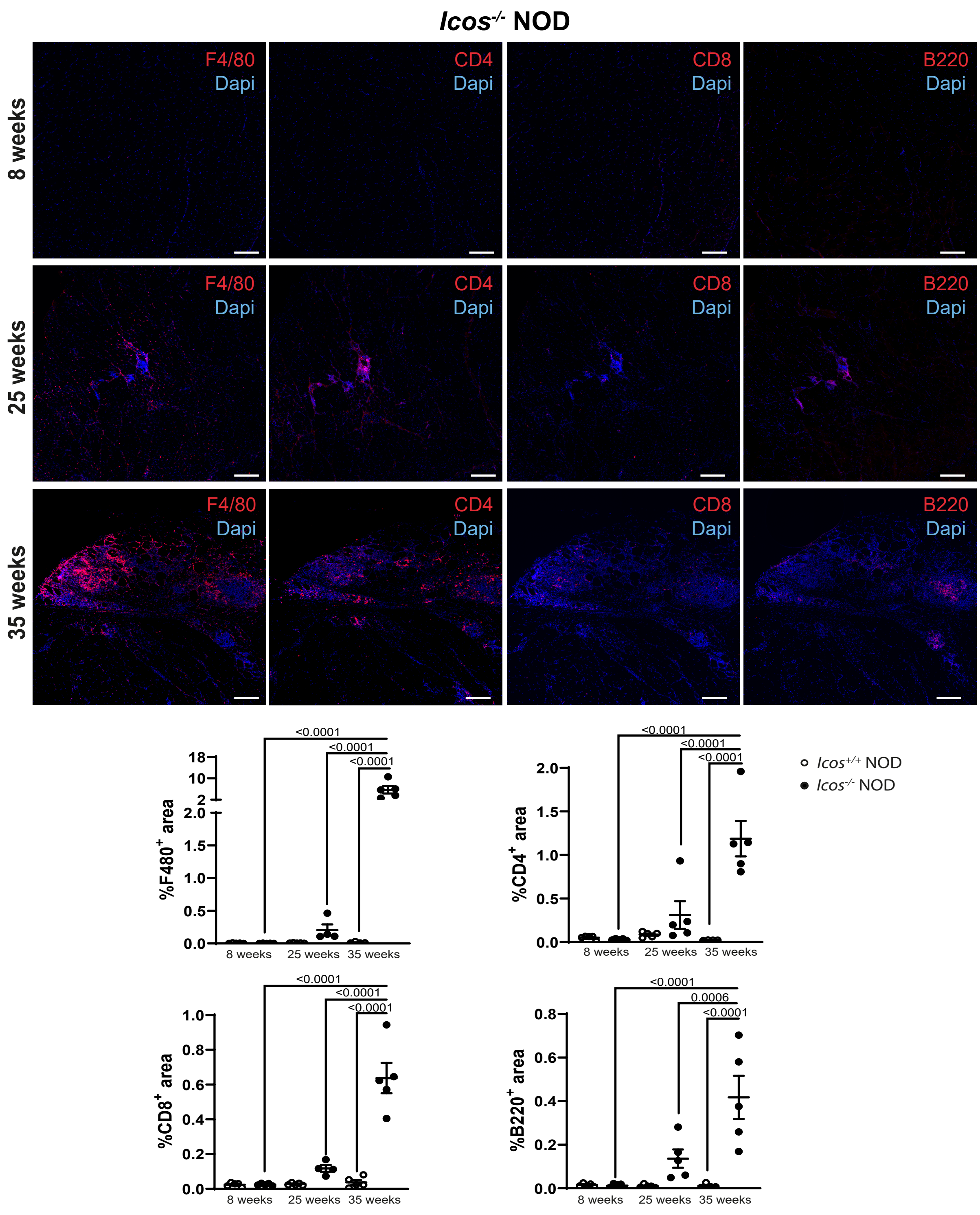

Supplementary Figure 1. Immunofluorescence labelling of immune cell populations infiltrating muscle sections of *Icos*$^{+/+}$ NOD and *Icos*$^{-/-}$ NOD mice of 8, 25 and 35 weeks of age. Immunofluorescence staining of muscle sections with antibodies against F4/80 (macrophages), CD4 (TCD4 lymphocytes), CD8a (TCD8 lymphocytes) or B220 (B cells). Images above are representative of *Icos*$^{-/-}$ NOD mice (n=5). Scale bars correspond to 200μm. Quantification of the immunoreactive area is shown below. Statistical analysis was performed using Two-way ANOVA (Interaction: F4/80: $F(2,23)= 15.26$, $p<0.0001$; CD4: $F(2,23)=15.62$, $p<0.0001$; CD8: $F(2,23)=37.06$, $p<0.0001$; B220: $F(2,23)=10.05$, $p=0.0007$) and Sidak's post hoc multiple comparison test (quantification corresponds to 2 sections/mouse from n=5 independent mice/group). Mean values ± s.e.m are shown. Source data are provided as a Source Data file.

a

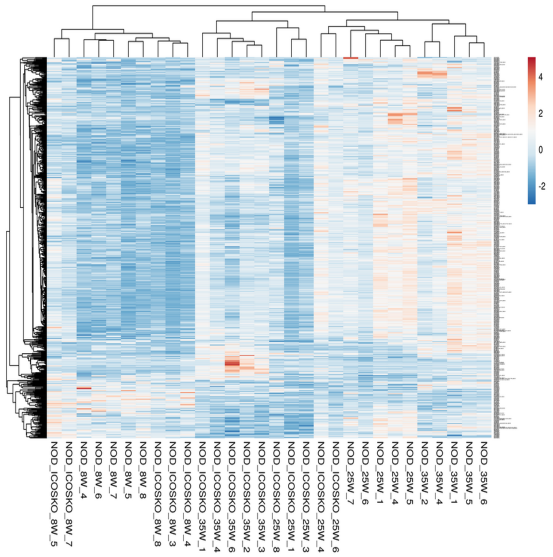

b

	Z score			p value		
**Canonical Pathways**	**8 weeks**	**25 weeks**	**35 weeks**	**8 weeks**	**25 weeks**	**35 weeks**
Oxidative Phosphorylation	-0.378	-4.583	-3.710	8.30E-06	4.02E-20	4.37E-20
LXR/RXR Activation	2.236	-2.449	-2.646	1.29E-03	2.64E-03	1.06E-05
TCA Cycle II (Eukaryotic)	-1.342	-2.000	-3.317	4.61E-07	1.54E-04	7.57E-16
Cardiac Hypertrophy Signaling	1.890	-2.828	-1.667	1.16E-03	6.25E-03	1.88E-03
Gluconeogenesis I	0.378	-2.828	-3.000	2.90E-10	3.28E-10	7.54E-12
Phospholipase C Signaling	1.134	-2.333	-2.530	1.67E-03	7.58E-12	1.91E-04
Glycolysis I	0.000	-3.000	-2.828	1.59E-08	7.28E-12	3.38E-10
Regulation of Actin-based Motility by Rho	2.000	-2.449	-1.134	3.57E-03	7.20E-04	1.02E-04
Fatty Acid β-oxidation I	0.000	-2.000	-3.000	5.78E-05	4.57E-04	6.38E-11
Gα12/13 Signaling	1.000	-2.236	-1.633	1.14E-02	1.71E-02	3.98E-03
PAK Signaling	1.000	-2.236	-1.633	4.15E-03	8.96E-04	1.33E-04
Sirtuin Signaling Pathway	0.816	2.828	1.225	4.17E-06	3.77E-13	5.96E-18
RhoGDI Signaling	-2.000	1.890	0.707	3.21E-02	1.09E-03	1.12E-03
Signaling by Rho Family GTPases	0.816	-2.646	-1.134	5.04E-03	1.99E-03	7.17E-03
CXCR4 Signaling	1.000	-1.633	-1.633	2.58E-02	1.26E-02	1.29E-02

c

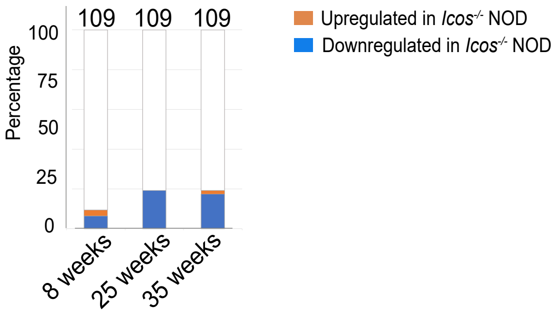

Supplementary Figure 2. Proteome analysis of muscles from *Icos*$^{+/+}$ NOD vs *Icos*$^{-/-}$ *NOD* mice of 8, 25 and 35 weeks of age additional data. a, Unsupervised hierarchical clustering of protein expression across all samples in the study (*Icos*$^{+/+}$ NOD and *Icos*$^{-/-}$ NOD mice of 8, 25 and 35 weeks of age). b, List of Top 15 IPA ‘Canonical pathways’ obtained after comparison of *Icos*$^{+/+}$ NOD vs *Icos*$^{-/-}$ NOD mice of 8, 25 and 35 weeks of age. Negative z-scores correspond to pathways downregulated in *Icos*$^{-/-}$ NOD mice, and positive z-scores correspond to pathways upregulated in *Icos*$^{-/-}$ NOD mice. c, Percentage of dysregulated proteins in *Icos*$^{-/-}$ NOD vs. *Icos*$^{+/+}$ NOD mice among all proteins of the IPA oxidative phosphorylation pathway (109 proteins). Data was obtained from n=5 mice/group.

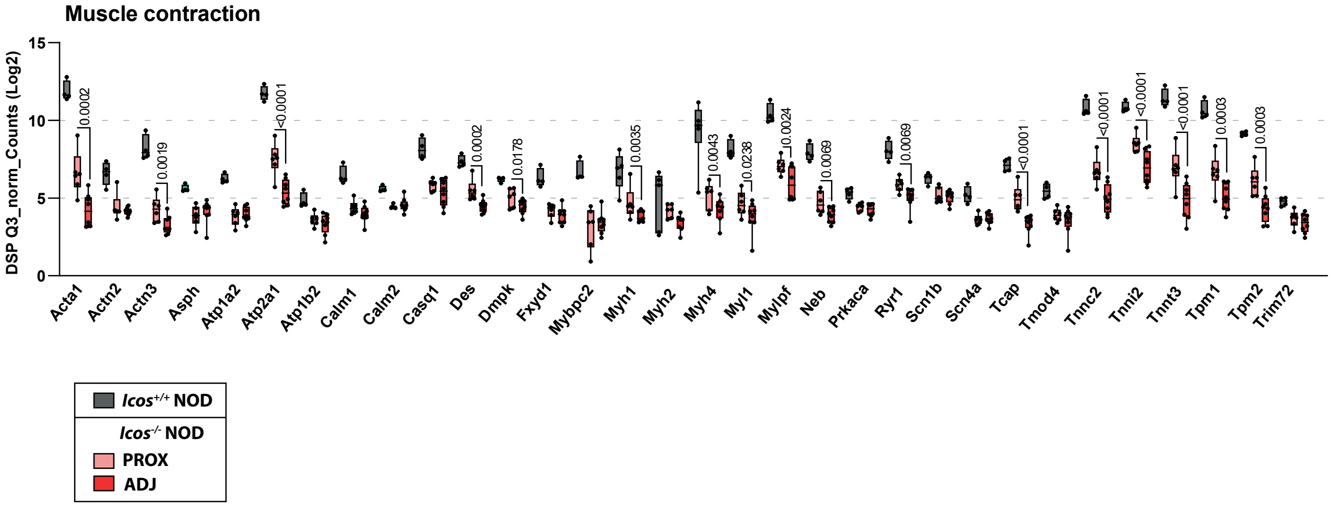

Supplementary Figure 3. mRNA expression of genes belonging to the category 'muscle contraction' in *Icos*$^{+/+}$ NOD and *Icos*$^{-/-}$ NOD PROX and ADJ. Histograms depicting normalized RNA counts for genes related to 'muscle contraction'. Box plots bounds to 25th to 75th percentiles, with line at the median, and whiskers expand from min to max values. mRNA counts for all genes in the histograms were statistically significantly higher in *Icos*$^{+/+}$ NOD (n=4) vs. *Icos*$^{-/-}$ NOD PROX (n=6) and ADJ (n=8) myofibers with p<0.05. Only *p* values (non adjusted) corresponding to *Icos*$^{-/-}$ NOD PROX vs. ADJ myofiber comparison are shown. Q3 normalization was used for all data and a Linear Mixed Model (LMM) was used for statistical analysis. *n* corresponds to the numbers of independent AOIs (areas of illumination). Source data are provided as a Source Data file.

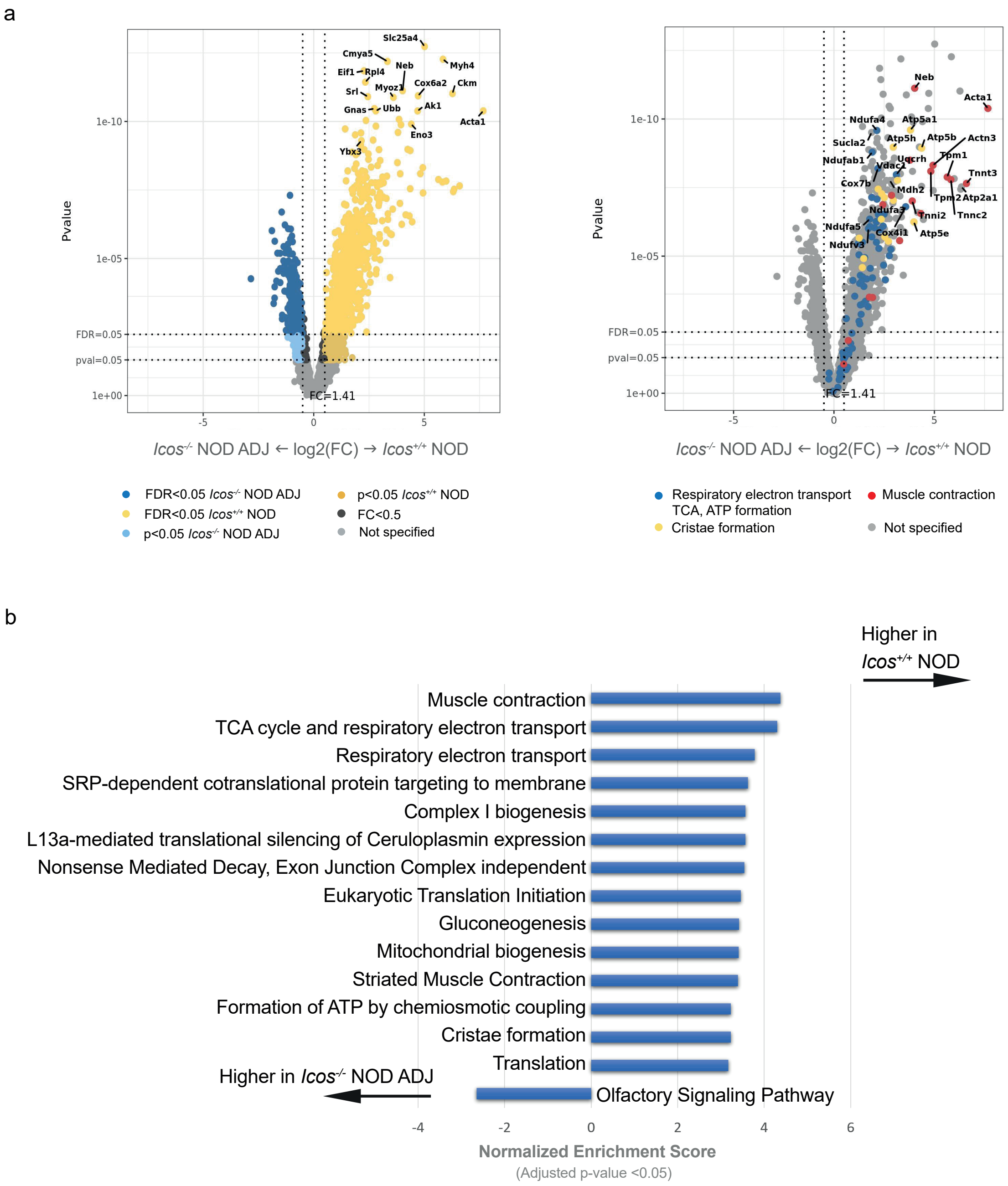

Supplementary Figure 4. Spatial transcriptome analysis of *Icos*$^{+/+}$ NOD vs. *Icos*$^{-/-}$ NOD ADJ myofibers. a, Volcano plots and b, pathway analysis from statistical comparisons between *Icos*$^{+/+}$ NOD (n=4) vs. *Icos*$^{-/-}$ NOD ADJ (n=8) myofibers. *n* corresponds to independent AOIs. Q3 normalization was used for all data and a Linear Mixed Model with Bonferroni-Hochberg (BH) correction was used for statistical analysis. For pathway analysis, Gene Set Enrichment Analysis (GEA) was performed. Source data are provided as a Source Data file.

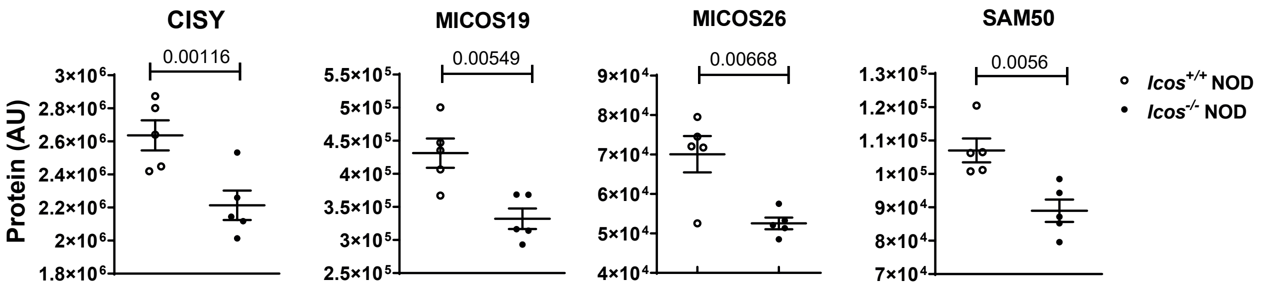

Supplementary Figure 5. Mitochondrial abundance/stability protein levels from proteome analysis. Levels of proteins related to mitochondrial abundance (citrate synthase; CISY) and Cristae stability (MICOS complex subunits MIC19, MIC26, and SAM50) in muscles from *Icos*$^{-/-}$ NOD vs. *Icos*$^{+/+}$ NOD mice at 35-weeks of age. Statistical analysis was performed using the inbuilt Progenesis statistical box called 'one-way ANOVA' (n=5 independent mice/ group). Mean values ± s.e.m are shown. *p* values are indicated in the graphs. Source data are provided as a Source Data file.

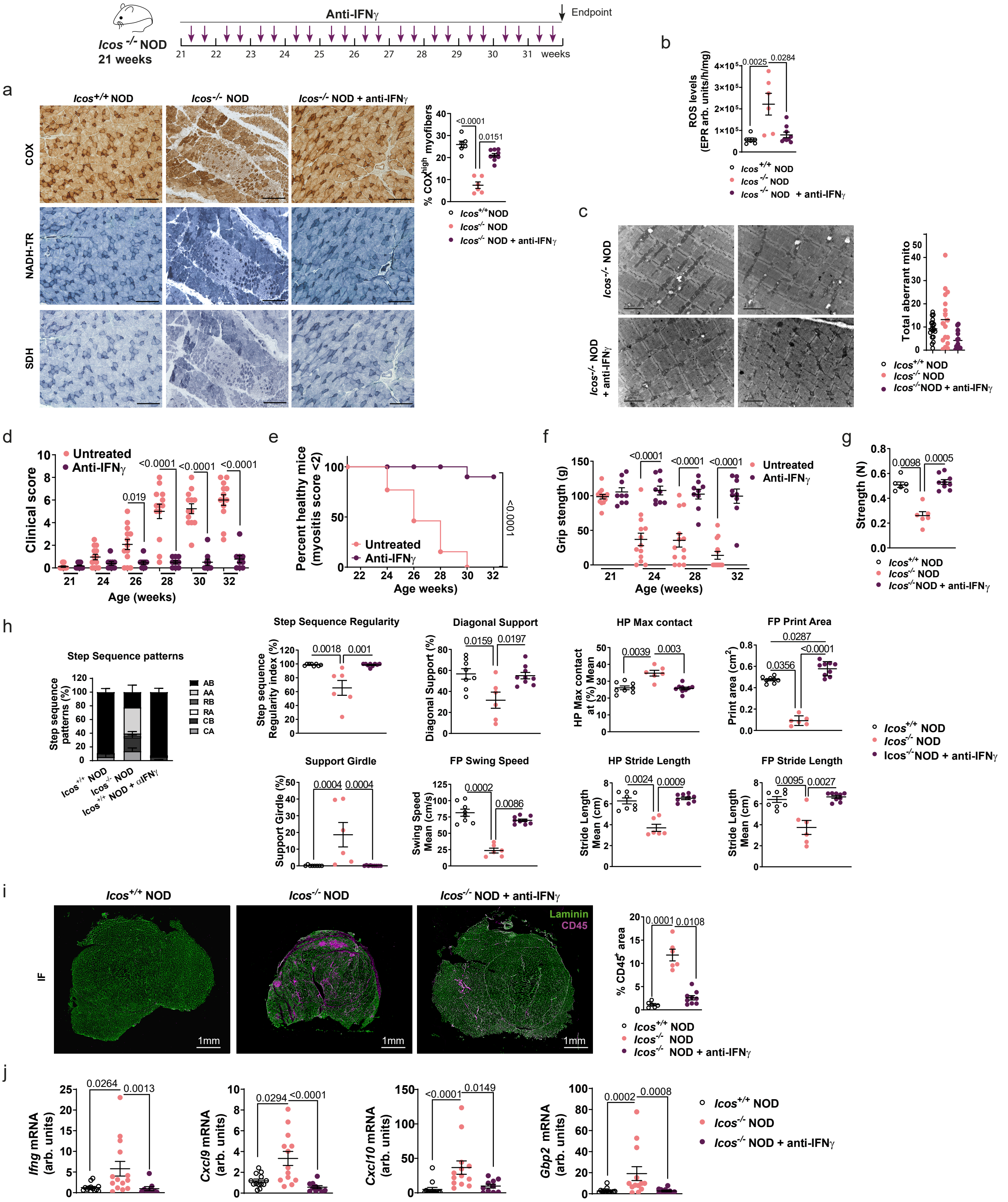

Supplementary Figure 6. Anti-IFNγ treatment ameliorates *Icos*$^{-/-}$ NOD myositis reducing oxidative stress and inflammation. *Icos*$^{-/-}$ NOD mice were treated twice per week with anti-IFNγ blocking antibody (200 µg/day, i.p.) starting at 21 until 32 weeks of age. a, COX, NADH-TR and SDH histoenzymology stainings with quantification of the percentage of COX^{high} fibers (scale bars, 200µm) (2 sections/mouse; n=6 for *Icos*$^{+/+}$ NOD mice and *Icos*$^{-/-}$ NOD mice, and n=9 for *Icos*$^{-/-}$ NOD + anti-IFNγ mice). b, EPR measurement of ROS production (n=6 for *Icos*$^{+/+}$ NOD and *Icos*$^{-/-}$ NOD mice and n=9 for *Icos*$^{-/-}$ NOD + anti-IFNγ mice). c, Electron microscopy images and quantification of aberrant state mitochondria (mitochondria in 15-20 fibers were quantified per mouse, with representative data from 1 mouse out of 2 being shown). Scale bars, 2µm. d, Clinical score. e, Percentage of disease-free mice. f, Grip strength. For d, e and f, n=13 *Icos*$^{-/-}$ NOD and n=9 *Icos*$^{-/-}$ NOD + anti-IFNγ mice. g, Muscle strength after sciatic nerve stimulation. h, Locomotor activity (Catwalk XT). For g and h, n=6 *Icos*$^{-/-}$ NOD mice and n=9 *Icos*$^{-/-}$ NOD + anti-IFNγ and n=6 (g) and n=8 (h) *Icos*$^{+/+}$ NOD mice. i, CD45/laminin immunofluorescence staining (scale bars, 1 mm). Graph depicts the mean CD45-immunoreactive area with respect to total muscle area (2 sections per mouse analysed with independent data for n=6 for *Icos*$^{+/+}$ NOD mice and *Icos*$^{-/-}$ NOD mice, and n=9 for *Icos*$^{-/-}$ NOD + anti-IFNγ). j, IFNγ-signature gene mRNA expression (arb. units: arbitrary units) with n=13 *Icos*$^{+/+}$ NOD and *Icos*$^{-/-}$ NOD mice and n=9 *Icos*$^{-/-}$ NOD + anti-IFNγ mice. Data corresponds to the mean ± s.e.m. For d and f, statistical analyses were performed by Two-Way ANOVA and Sidak's post-hoc test. For e, Log-rank test DF: 1. For a, g, h, i, the Kruskal-Wallis test with Dunn's test (uncorrected) was used. All *n* correspond to independent mice. Mean values ± s.e.m and *p* values are shown. Data from one representative experiment out of two is shown. Source data are provided as a Source Data file.

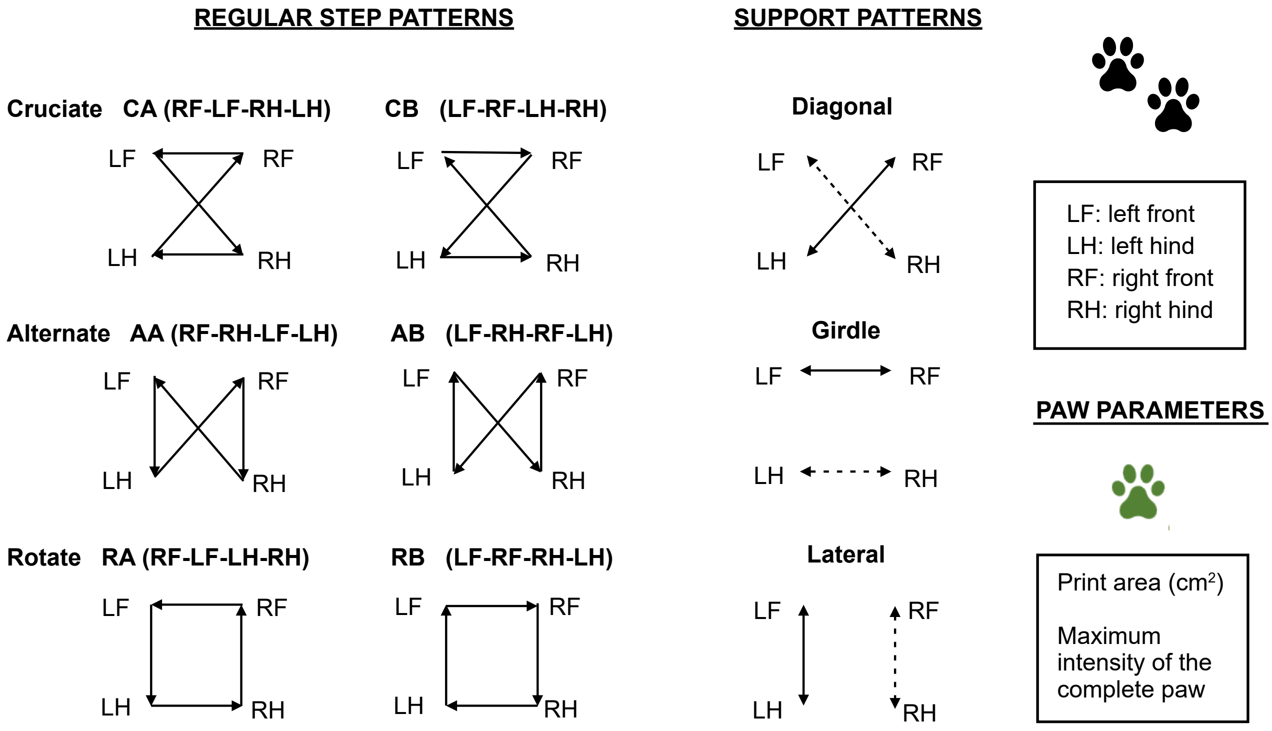

STEP SEQUENCE REGULARITY INDEX

Number of normal step sequence patterns relative to the total number of paw placements (fractional measure of inter-paw coordination).

PHASE DISPERSIONS

Temporal relationship between placement of two paws within a Step cycle (time between two consecutive initial contacts of the same paw). Measure of inter-paw coordination.

DUTY CYCLE (%)

Duty cycle= Ratio stand time/step cycle.
Stand time (s): duration of contact with the walkway of a specific paw.
Step cycle (s): duration of two consecutive initial contacts of a specific paw.

SWING SPEED

Swing speed (cm/s): speed of the paw during swing (no contact of a paw with the glass plate).

Supplementary Figure 7. Description of Catwalk parameters. Graphical representation of selected gait parameters. Each of the four different paws of a given mouse were referred to as RF (right front paw), LF (left front paw), RH (right hind paw) and LH (left hind paw). The CatWalk XT (version 10.6) gait analysis system (Noldus, Netherlands system) consisted of a 1.3-m black corridor on a glass plate with a green LED lit inside, located in a dark and silent room. By means of Illuminated footprints technology, paws were captured by a high-speed video camera (100 frames per second) positioned underneath the glass. A compliant run was considered when a mouse walked across the runway without stopping, turning around, or changing direction. At least three compliant runs were necessary to achieve a completed testing run.

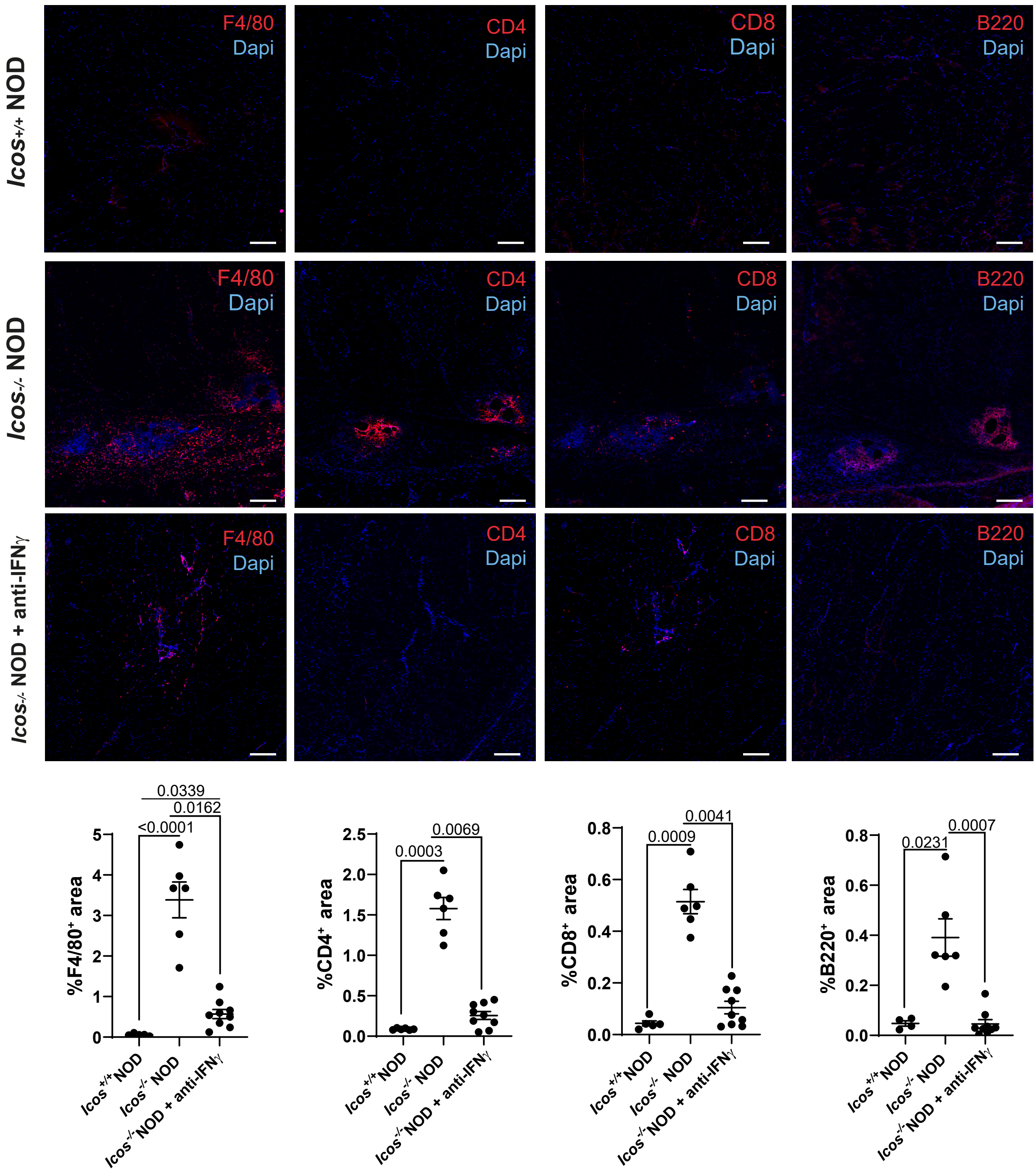

Supplementary Figure 8. Immunofluorescence labelling of immune cell populations infiltrating muscle sections of *Icos*$^{-/-}$ NOD upon treatment with anti-IFNγ antibodies. *Icos*$^{-/-}$ NOD mice were treated twice per week with anti-IFNγ blocking antibody (200 µg/day, i.p.) starting at 21 weeks until 32 weeks of age. Immunofluorescence staining of muscle infiltrates with antibodies against F4/80 (macrophages), CD4 (TCD4 lymphocytes), CD8a (TCD8 lymphocytes) or B220 (B cells). Scale bars correspond to 200µm. Quantification of the immunoreactive area is shown below. Statistical analysis was performed using Kruskal-Wallis test (quantification corresponds to 2 sections/mouse from n=5 *Icos*$^{+/+}$ NOD mice, n=6 *Icos*$^{-/-}$ NOD mice and n=9 *Icos*$^{-/-}$ NOD + anti IFNγ mice/group). For all, *n* corresponds to independent mice. Mean values ± s.e.m and *p* values are shown. Data from one representative experiment out of two is shown. Source data are provided as a Source Data file.

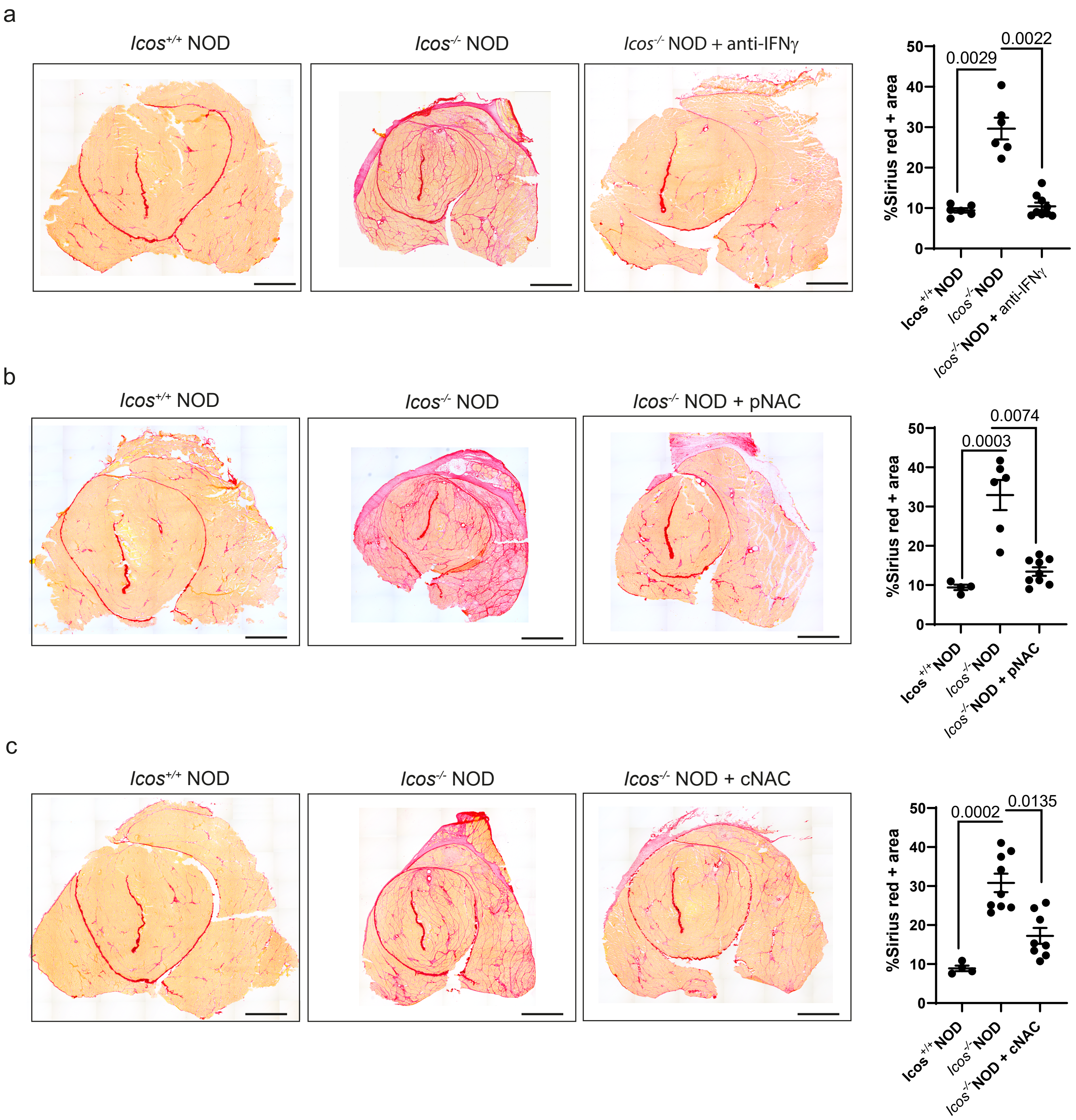

Supplementary Figure 9. Fibrosis analysis in *Icos*$^{-/-}$ NOD mice. Sirius red staining of muscle sections from *Icos*$^{+/+}$ NOD mice, *Icos*$^{-/-}$ NOD mice and *Icos*$^{-/-}$ NOD mice treated with a, anti-IFNγ (n=6 *Icos*$^{+/+}$ NOD mice and *Icos*$^{-/-}$ NOD mice, and n=9 *Icos*$^{-/-}$ NOD + anti-IFNγ), b, preventive NAC (pNAC, n=4 *Icos*$^{+/+}$ NOD mice, n=6 *Icos*$^{-/-}$ NOD mice, and n=9 *Icos*$^{-/-}$ NOD + NAC) and c, curative NAC (cNAC, n=4 *Icos*$^{+/+}$ NOD mice, n=9 *Icos*$^{-/-}$ NOD mice, and n=8 *Icos*$^{-/-}$ NOD + NAC) with *n* corresponding to independent mice. Quantification corresponds to 2 sections/mouse. Scale bars correspond to 1mm. Statistical analysis was performed by Kruskal-Wallis test. Data from one representative experiment out of two is shown. Mean values and s.e.m. are shown. Source data are provided as a Source Data file.

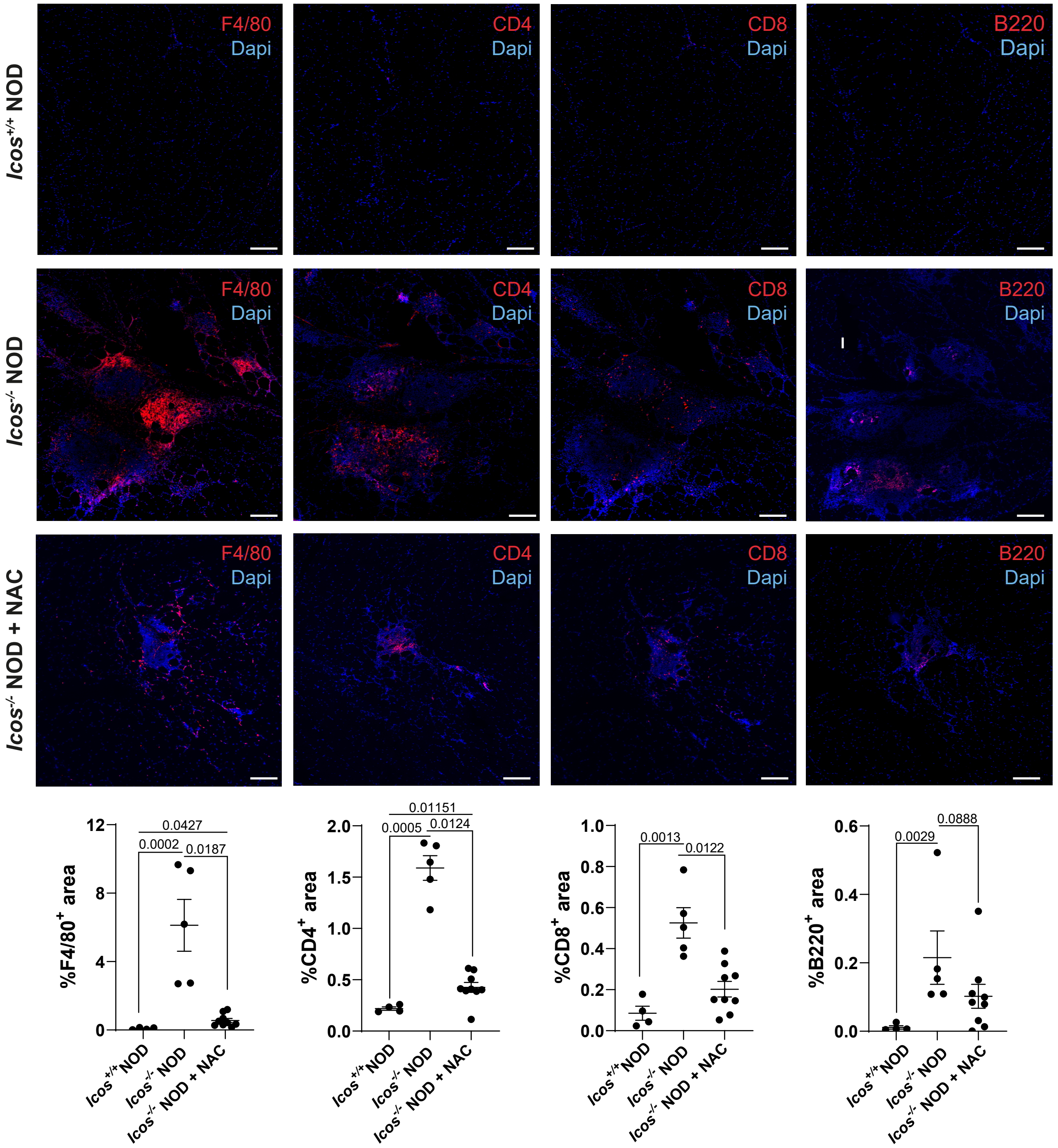

Supplementary Figure 10. Immunofluorescence labelling of immune cell populations infiltrating muscle sections of *Icos*$^{-/-}$ NOD upon treated with NAC in preventive setting. *Icos*$^{-/-}$ NOD mice were treated with NAC (2g/L) starting at 14 weeks of age until 32 weeks of age. Immunofluorescence staining of muscle infiltrates with antibodies against F4/80 (macrophages), CD4 (TCD4 lymphocytes), CD8a (TCD8 lymphocytes) or B220 (B cells). Scale bars correspond to 200μm. Quantification of the immunoreactive area is shown below. Quantification of the immunoreactive area is shown below. Statistical analysis was performed using Kruskal-Wallis test (quantification corresponds to 2 sections/mouse from n=4 *Icos*$^{+/+}$ NOD mice, n=5 *Icos*$^{-/-}$ NOD mice and n=9 *Icos*$^{-/-}$ NOD + NAC mice/group, with *n* corresponding to independent mice). Mean values ± s.e.m and *p* values are shown. Data from one representative experiment out of two is shown. Source data are provided as a Source Data file.

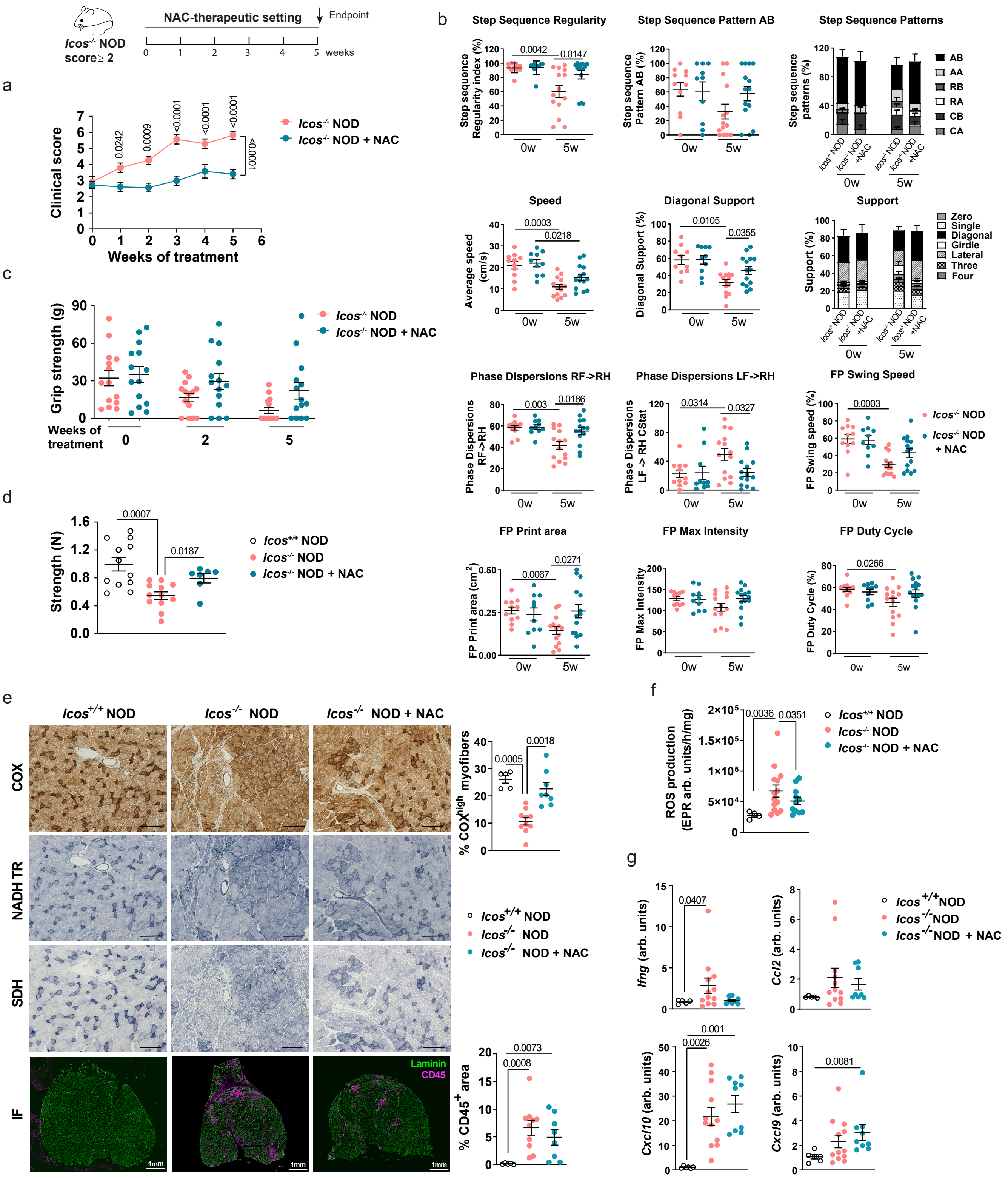

Supplementary Figure 11. Curative NAC treatment hampers *Icos$^{-/-}$* NOD mice myositis progression. *Icos$^{-/-}$* NOD mice reaching a clinical score of 2 were treated for five weeks with NAC (2 g/L). a, Clinical score. b, Locotomor activity (Catwalk XT). c, Grip strength. d, Muscle strength after sciatic nerve stimulation. e, Histopathological analysis. COX, NADH-TR and SDH histoenzymology stainings with quantification of the percentage of COX^{high} fibers(scale bars, 200 µm) and CD45/laminin immunofluorescence staining with quantification of CD45-immunoreactive area with respect to total muscle area (scale bars, 1mm) . f, EPR measurement of ROS production. g, Chemokine and cytokine mRNA expressions (arb. units: arbitrary units). Data corresponds to the mean values ± s.e.m. For a and c, n=14 mice/ group. For b, n=11 *Icos$^{-/-}$* NOD and n=10 *Icos$^{-/-}$* NOD + NAC mice at 0 weeks, and n=14 mice/group at 5 weeks. For d, n=12 for *Icos$^{+/+}$* NOD and *Icos$^{-/-}$* NOD mice, and n=7 *Icos$^{-/-}$* NOD + NAC mice. For histological quantification analyses, data corresponds to 2 sections/mouse from n=5 *Icos$^{+/+}$* NOD mice, n=10 *Icos$^{-/-}$* NOD mice and n=9 *Icos$^{-/-}$* NOD + NAC mice/group. For cytokine expression analysis, n=5 *Icos$^{+/+}$* NOD mice, n=12 *Icos$^{-/-}$* NOD mice and n=9 *Icos$^{-/-}$* NOD + NAC mice/group. For a and c, comparisons were performed by Two-way ANOVA and Sidak's multiple comparison post hoc test. For b, d, e, f, and g, with statistical analyses performed using the Kruskal-Wallis test with Dunn's (uncorrected) multiple comparison test. *p* values are shown. For all, *n* corresponds to independent mice. A representative experiment out of two is shown. Source data are provided as a Source Data file.

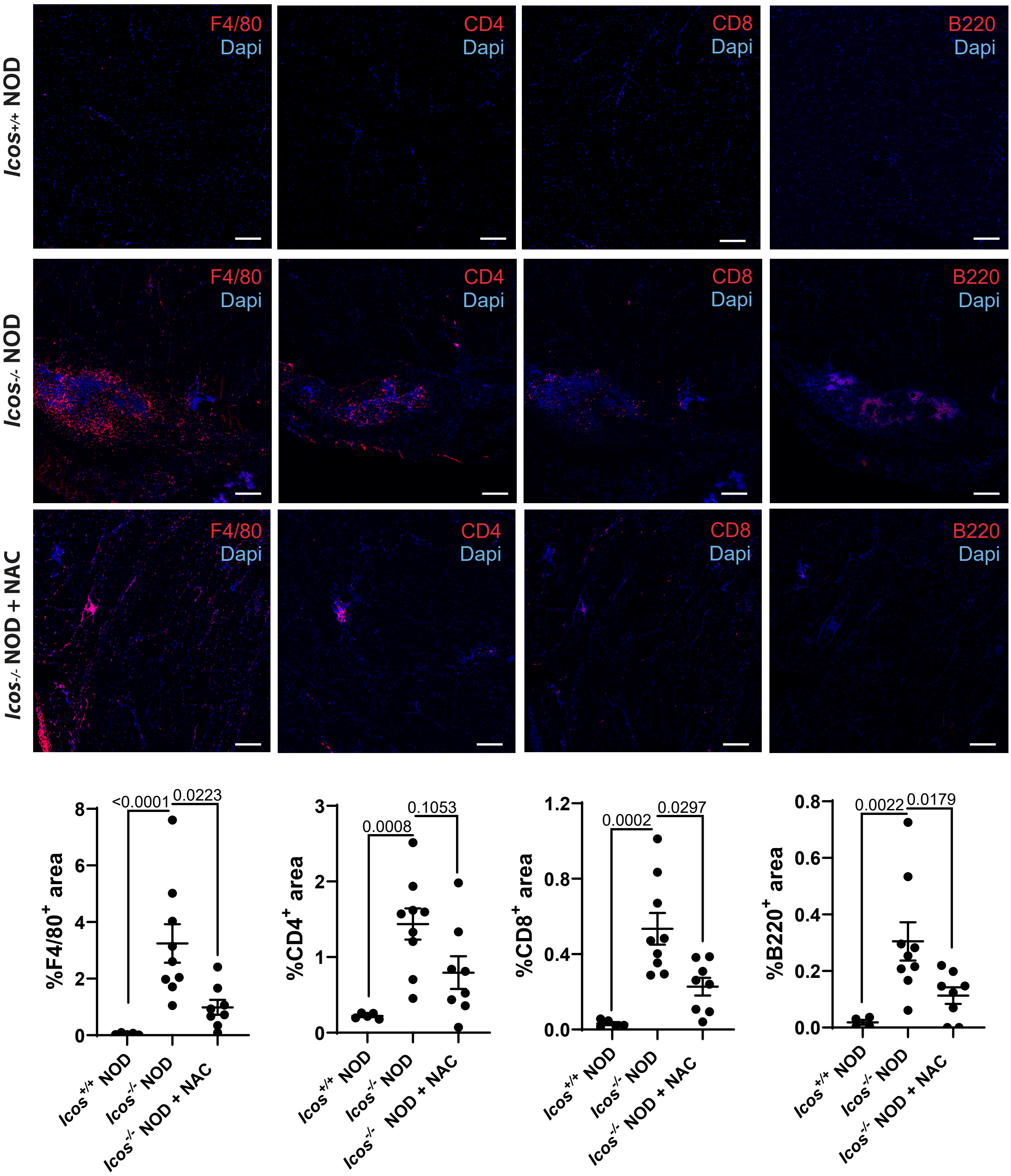

Supplementary Figure 12. Immunofluorescence labelling of immune cell populations infiltrating muscle sections of *Icos*$^{-/-}$ NOD upon treated with NAC in curative setting. *Icos*$^{-/-}$ NOD mice reaching a clinical score 2 were treated with NAC (2g/L) for 5 weeks. Immunofluorescence staining of muscle infiltrates with antibodies against F4/80 (macrophages), CD4 (TCD4 lymphocytes), CD8a (TCD8 lymphocytes) or B220 (B cells). Scale bars correspond to 200µm. Quantification of the immunoreactive area is shown below. Quantification of the immunoreactive area is shown below. Statistical analysis was performed using Kruskal-Wallis test and Dunn's (uncorrected) post-hoc test (quantification corresponds to 2 sections/mouse from n=5 *Icos*$^{+/+}$ NOD mice, n=9 *Icos*$^{-/-}$NOD mice and n=8 *Icos*$^{-/-}$ NOD + NAC mice/group). For all, *n* corresponds to independent mice. Mean values ± s.e.m and *p* values are shown. Data corresponds to one representative experiment out of two. Source data are provided as a Source Data file.

a

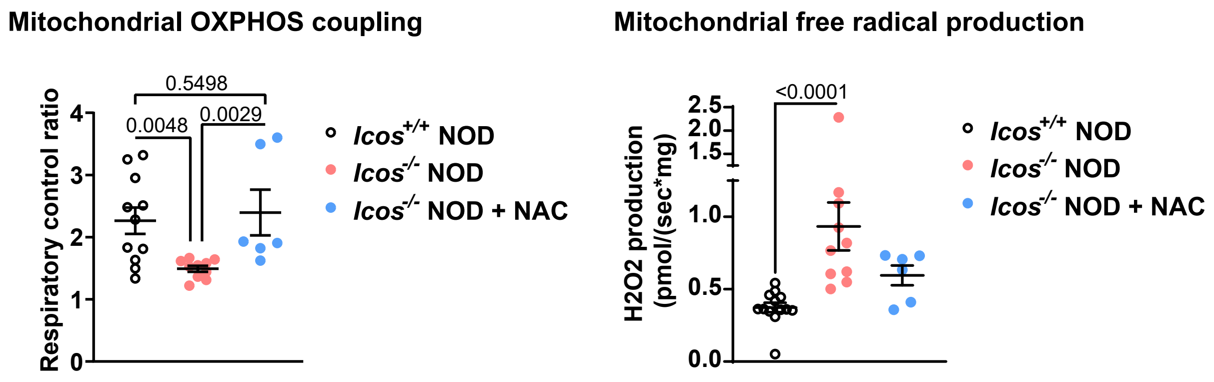

b

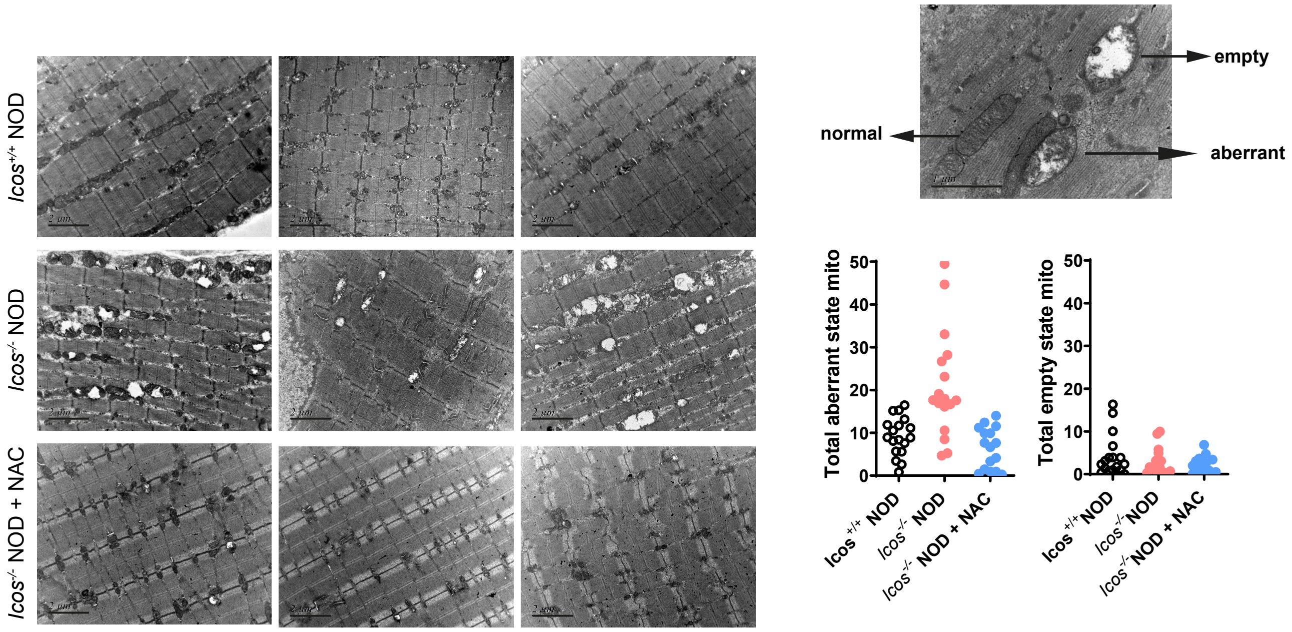

Supplementary Figure 13. NAC treatment improves *Icos*$^{-/-}$ NOD mice mitochondrial functional and morphological features. *Icos*$^{-/-}$ NOD mice were treated with NAC (2 g/L) starting when reaching a clinical score of 2 and lasting for five weeks. a, *Ex vivo* analysis of mitochondrial respiratory function (respiratory control ratio) and H_2O_2 production in the presence of succinate in muscles from *Icos*$^{+/+}$ NOD mice, *Icos*$^{-/-}$ NOD mice and *Icos*$^{-/-}$ NOD mice treated with NAC (for OXPHOS coupling, *Icos*$^{+/+}$ NOD and *Icos*$^{-/-}$ NOD n=11 mice/group, for NAC-treated mice n=6; for H_2O_2, *Icos*$^{+/+}$ NOD n=12, *Icos*$^{-/-}$ NOD n=10 and for NAC-treated, n=6). b, Electron microscopy representative images of muscles from *Icos*$^{+/+}$ NOD mice, *Icos*$^{-/-}$ NOD mice and *Icos*$^{-/-}$ NOD mice treated with NAC. Scale bars of left panels correspond to 2μm. Scale bar on the right image (top, higher magnification) corresponds to 1μm. Graph depicts quantification of aberrant and empty mitochondria. Each dot corresponds to the mean quantification of 15-20 microscopic fields per myofiber, with 16-18 myofibers being studied per mouse. For a, results correspond to independent mice from one experiment. Mean values ± s.e.m and *p* values are shown. For b, representative results for one mouse out of two are shown. Statistical analyses were performed using Kruskal Wallis and uncorrected Dunn's test. Mean and s.e.m are shown. Source data are provided as a Source Data file.

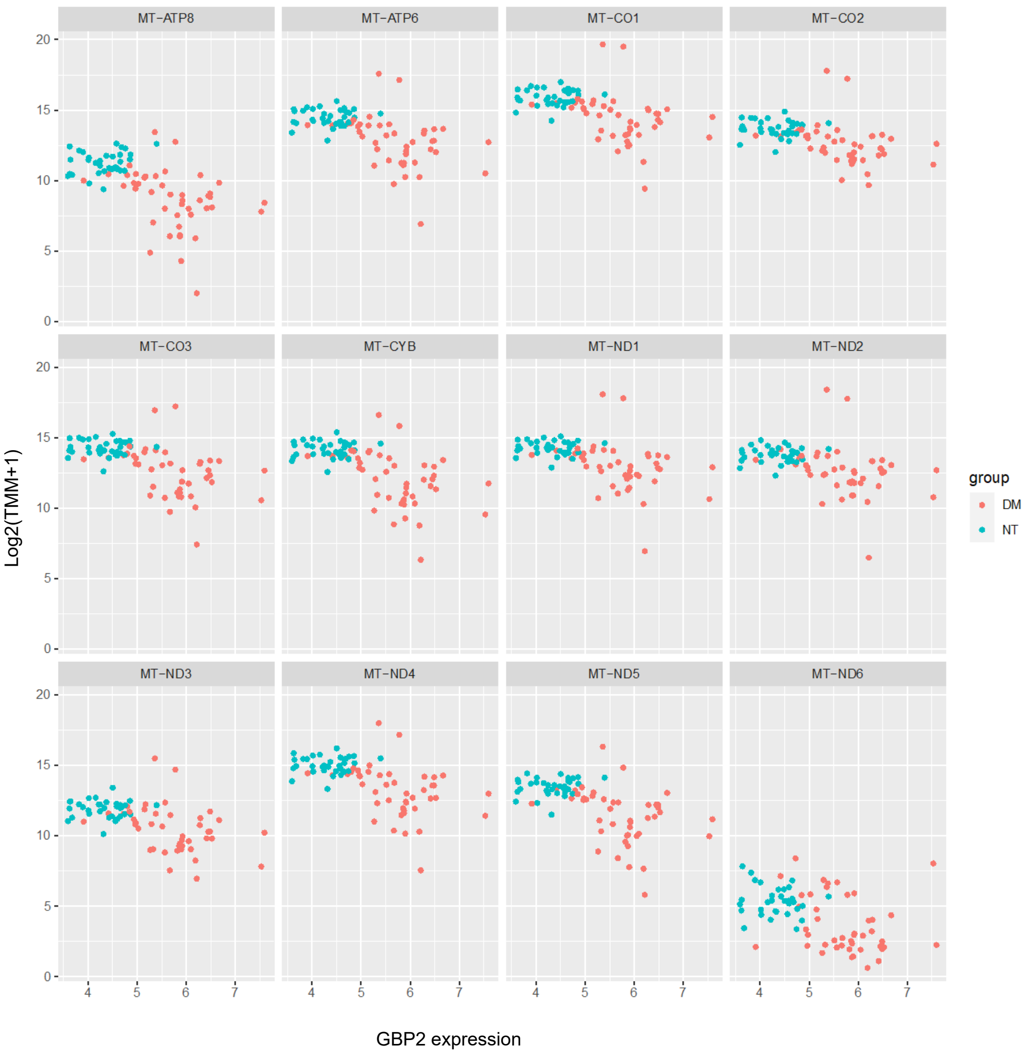

Supplementary Figure 14. Correlation of GBP and mitochondrial OXPHOS gene expression in human DM. Analysis of bulk transcriptomic data of muscle biopsies from 44 DM patients tested positive for myositis-specific autoantibodies against NXP2 (n = 14), TIF1γ (n = 12), Mi2 (n = 12) and MDA5 (n = 6), and from 33 histologically normal muscle biopsies focusing on mitochondrial genes. NT, normal tissue; DM, dermatomyositis. A strong correlation of the expression of these mitochondrial genes with GBP can be observed. Trimmed Means of M values (TMM). For all, *n* corresponds to independent subjects. Source data are provided as a Source Data file.

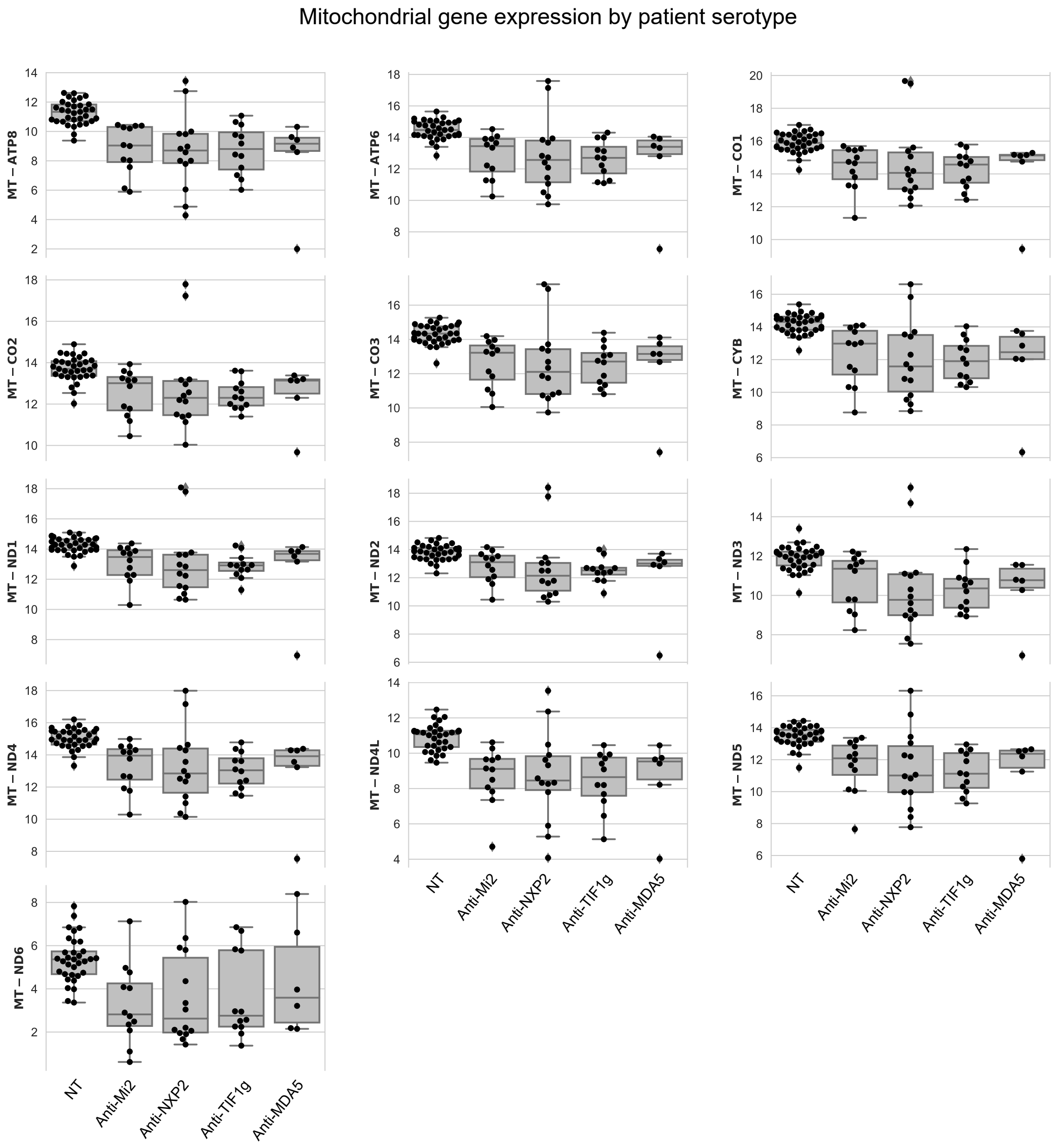

Supplementary Figure 15. Expression of mitochondrial genes by myositis patient serotype. Analysis of bulk transcriptomic data of muscle biopsies from 44 DM patients tested positive for myositis-specific autoantibodies against NXP2 (n = 14), TIF1γ (n = 12), Mi2 (n = 12) and MDA5 (n = 6), and from 33 histologically normal muscle biopsies focusing on mitochondrial genes. Data was classified by patient serotype. NT, normal tissue. The Benjami-ni–Hochberg correction was used to adjust for multiple comparisons, and a corrected value of *p* (*q* value) ≤ 0.05 was considered statistically significant. The Benjamini–Hochberg correction was used to adjust for multiple comparisons. Box plots bounds to 25th to 75th percentiles, with line at the median, and whiskers correspond to 1.5 times the inter-quantile range (1.5x[Q3-Q1]). For all, *n* corresponds to independent subjects. Source data are provided as a Source Data file.

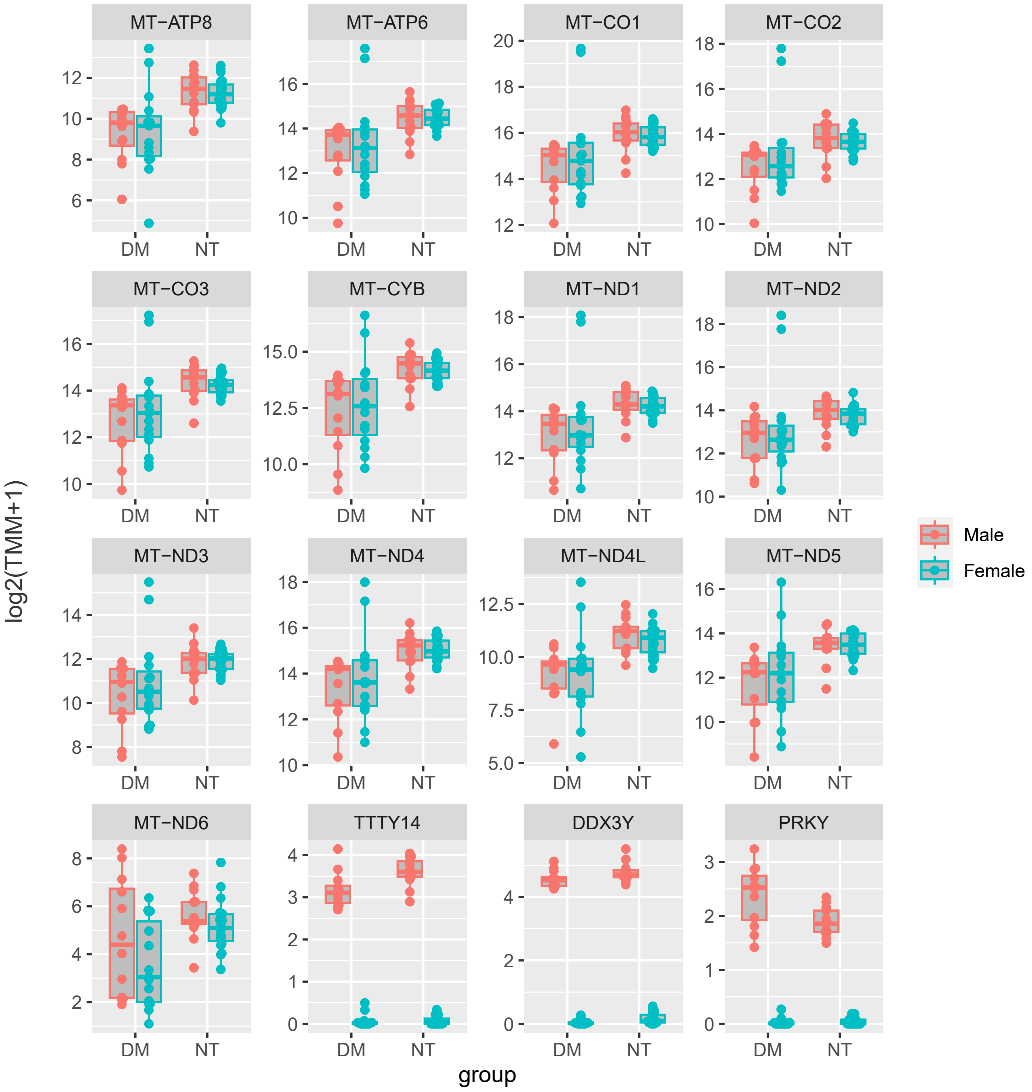

Supplementary Figure 16. Expression of mitochondrial genes by myositis patient sex. Analysis of bulk transcriptomic data of muscle biopsies from 44 DM patients tested positive for myositis-specific autoantibodies against NXP2 (n = 14), TIF1γ (n = 12), Mi2 (n = 12) and MDA5 (n = 6), and from 33 histologically normal muscle biopsies focusing on mitochondrial genes. Data was classified by patient sex. NT, normal tissue; DM, dermatomyositis. Box plots bounds to 25th to 75th percentiles, with line at the median, and whiskers correspond to 1.5 times the interquantile range (1.5x[Q3-Q1]). For all, *n* corresponds to independent subjects. The Benjamini–Hochberg was used to adjust for multiple comparisons. Source data are provided as a Source Data file.

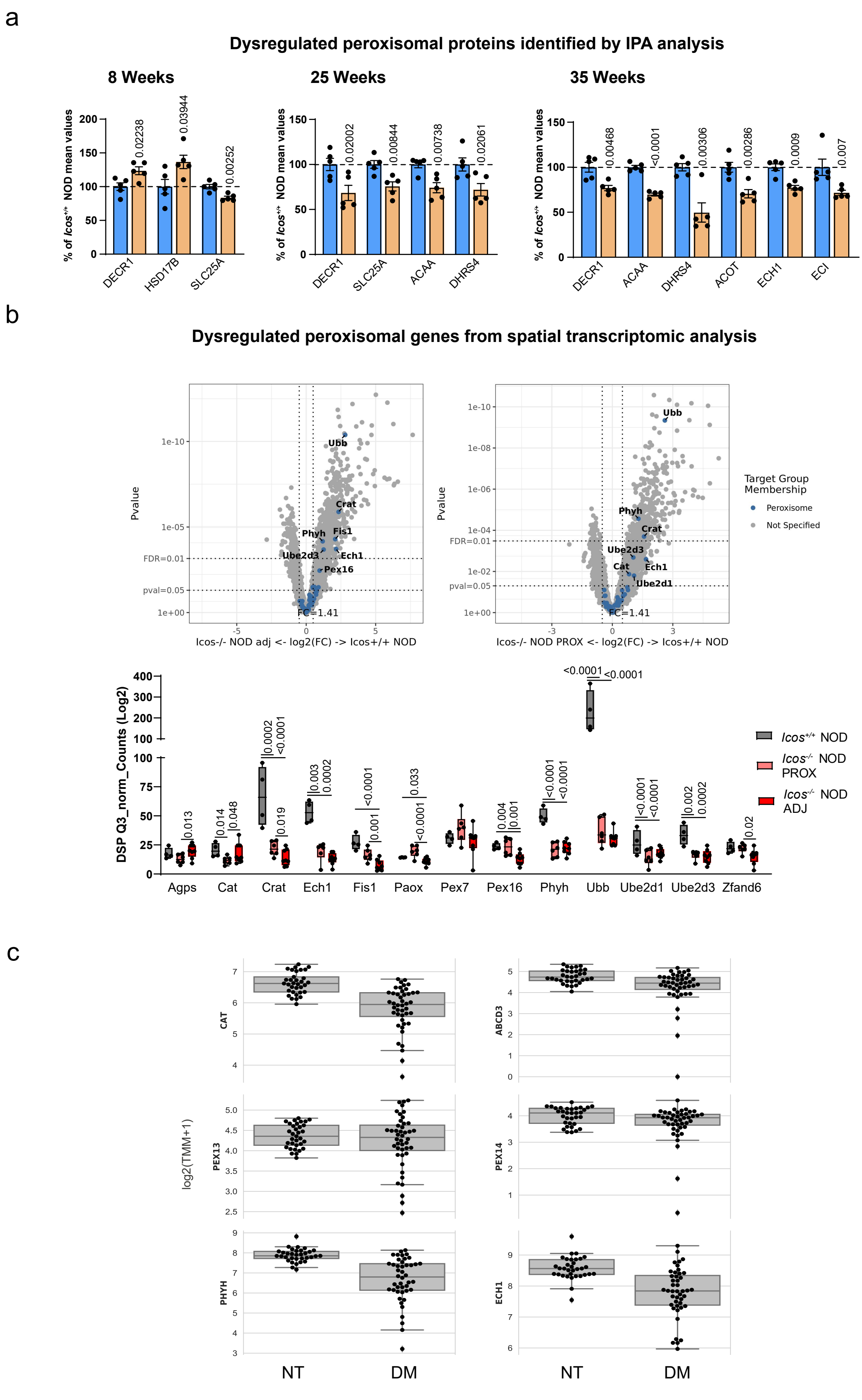

Supplementary Figure 17. Analysis of proteome and transcriptome data focusing on peroxisome. a, Proteome analysis of *Icos*$^{+/+}$ NOD and *Icos*$^{-/-}$ NOD mice of 8, 25 and 35 weeks of age (n=5/group). Graph represents the significantly dysregulated proteins identified by IPA as belonging to the category "Peroxisome". Mean values and s.e.m. as well as p values calculated with Progenesis analysis box ANOVA with raw data are shown. b, Volcano plots highlighting the genes belonging to the peroxisome category (blue) in *Icos*$^{+/+}$ NOD (n=4) vs. *Icos*$^{-/-}$ NOD ADJ (n=8) or PROX (n=6) AOI. Graph below illustrates the expression levels of peroxisome genes. Statistical analysis was performed using a Linear Mixed Model (LMM) with Bonferroni-Hochberg (BH) correction. Non adjusted p values are shown. Box plots bounds to 25th to 75th percentiles, with line at the median, and whiskers expand from min to max values. c, Analysis of bulk transcriptomic data of muscle biopsies from 44 DM and from 33 histologically normal muscle biopsies. Box plots bounds to 25th to 75th percentiles, with line at the median, and whiskers correspond to 1.5 times the interquantile range (1.5x[Q3-Q1]). The Benjamini–Hochberg correction was used to adjust for multiple comparisons, and a corrected value of *p* (*q* value) ≤ 0.05 was considered statistically significant. For panels a and b, *n* represents independent mice. For c, *n* represents independent subjects. Source data are provided as a Source Data file.

Supplementary Table 1. Seropositivity for anti-TNNT3 antibodies in *Icos*$^{-/-}$ NOD mice

**Preventive NAC (pNAC)**	**Anti-TNNT3 Ab**		The chi-square statistic is 4.1771. The *p*-value is .040974. The result is significant at $p < .05$.
	**+**	**-**	
Icos$^{-/-}$ NOD (n=21)	11	10	
Icos$^{-/-}$ NOD + pNAC (n=19)	4	15	
**Curative NAC (cNAC)**	**Anti-TNNT3 Ab**		The chi-square statistic is 0.9. The *p*-value is .342782. The result is *not* significant at $p < .05$.
	+	-	
Icos$^{-/-}$ NOD (n=16)	12	4	
Icos$^{-/-}$ NOD + cNAC (n=20)	12	8	

Supplementary Table 1. Effect of NAC treatment on the levels of anti-TNNT3 antibodies. Anti-TNNT3 antibodies were detected by addressable laser bead immunoassay (ALBIA) as described in[19] and as described in the material and methods section. Samples represent cumulative data of two independent experiments (Ab: antibody). For statistic analysis, a chi-square test was used. Source data are provided as a Source Data file.

Supplementary Table 2. Supplementary information of NT vs DM mitochondrial gene expression analysis

	gene_id	logFC	AveExpr	t	*p*.Value	adj.*p*.Val	B
1	*MT-ATP6*	-1.5183541	13.426885	-4.023346	1.30E-04	6.37E-04	0.8570132
2	*MT-ATP8*	-2.4910282	9.721537	-5.62142	2.70E-07	3.93E-06	6.5565431
3	*MT-CO1*	-1.2557334	14.999692	-3.533374	6.86E-04	2.61E-03	-0.618753
4	*MT-CO2*	-0.8626444	13.017849	-2.787003	6.65E-03	1.75E-02	-2.7632536
5	*MT-CO3*	-1.498221	13.246799	-4.15192	8.21E-05	4.33E-04	1.2737775
6	*MT-CYB*	-1.9417794	12.906368	-4.861908	5.74E-06	4.68E-05	3.7556536
7	*MT-ND1*	-1.0837725	13.466636	-3.001681	3.58E-03	1.05E-02	-2.1801165
8	*MT-ND2*	-0.9317278	13.071268	-2.477466	1.53E-02	3.54E-02	-3.4957444
9	*MT-ND3*	-1.223644	11.010032	-3.607247	5.38E-04	2.11E-03	-0.5991156
10	*MT-ND4*	-1.6431345	13.922508	-4.396597	3.36E-05	2.02E-04	2.1298788
11	*MT-ND4L*	-2.15401	9.549232	-5.447703	5.52E-07	6.91E-06	5.8574816
12	*MT-ND5*	-1.8625561	12.243666	-4.708456	1.04E-05	7.60E-05	3.172838
13	*MT-ND6*	-2.0950477	4.162208	-5.544	3.72E-07	5.09E-06	6.161829

Supplementary Table 2. Supplementary information of NT vs DM mitochondrial gene expression analysis. Analysis of bulk transcriptomic data of muscle biopsies from 44 DM patients tested positive for myositis-specific autoantibodies against NXP2 (n = 14), TIF1γ (n = 12), Mi2 (n = 12) and MDA5 (n = 6), and from 33 histologically normal muscle biopsies focusing on mitochondrial genes (*ATP8*, *ATP6*, *CO1*, *CO2*, *CO3*, *ND3*, *ND4*, *ND4L*, *ND5* and *ND6*) with *n* corresponding to individual subjects. The table shows statistical analysis details of comparison between DM and normal samples. The Benjamini–Hochberg was used to adjust for multiple comparisons, and a corrected value of *p* (*q* value) ≤ 0.05 was considered statistically significant. Source data are provided as a Source Data file.

Supplementary Table 3. Spearman correlation *p*-values

	GBP2	IFI30	IFNG	CD3E	CD4	CD8A	CD14	CD68	NCAM1	MYOG	PAX7	MYH3	MYH8	ACTA1	MYH1	MYH2
MT-ATP8	0.0019	0.0000	0.0097	0.0092	0.1609	0.3056	0.0003	0.0004	0.0061	0.0000	0.2427	0.0003	0.2255	0.0035	0.0389	0.2270
MT-ATP6	0.0008	0.0000	0.0170	0.0136	0.1111	0.2298	0.0003	0.0003	0.0063	0.0003	0.3107	0.0002	0.2277	0.0016	0.0325	0.2515
MT-CO1	0.0008	0.0000	0.0119	0.0190	0.1426	0.2560	0.0002	0.0002	0.0081	0.0004	0.5219	0.0008	0.3194	0.0005	0.0295	0.1982
MT-CO2	0.0012	0.0005	0.0304	0.0131	0.1698	0.1930	0.0012	0.0011	0.0034	0.0007	0.4193	0.0008	0.2119	0.0035	0.0551	0.4000
MT-CO3	0.0010	0.0000	0.0285	0.0178	0.1556	0.2659	0.0003	0.0002	0.0039	0.0002	0.3715	0.0003	0.3405	0.0011	0.0327	0.2317
MT-CYB	0.0006	0.0000	0.0148	0.0113	0.1047	0.2081	0.0001	0.0001	0.0029	0.0002	0.2752	0.0000	0.2507	0.0008	0.0235	0.2008
MT-ND1	0.0035	0.0002	0.0102	0.0118	0.1137	0.1600	0.0003	0.0002	0.0094	0.0017	0.6166	0.0018	0.3079	0.0007	0.0632	0.2902
MT-ND2	0.0111	0.0007	0.0307	0.0322	0.2353	0.2797	0.0011	0.0012	0.0164	0.0050	0.7364	0.0067	0.5487	0.0026	0.1109	0.4424
MT-ND3	0.0060	0.0006	0.0636	0.0600	0.2696	0.4277	0.0015	0.0009	0.0053	0.0005	0.5699	0.0017	0.5139	0.0058	0.1086	0.4517
MT-ND4	0.0006	0.0000	0.0136	0.0164	0.0810	0.2434	0.0001	0.0001	0.0058	0.0003	0.4106	0.0003	0.2153	0.0009	0.0204	0.2704
MT-ND4L	0.0010	0.0000	0.0056	0.0227	0.1654	0.3948	0.0001	0.0002	0.0104	0.0000	0.2520	0.0002	0.2839	0.0030	0.0259	0.2324
MT-ND5	0.0017	0.0000	0.0158	0.0370	0.1778	0.4051	0.0003	0.0002	0.0120	0.0005	0.4111	0.0003	0.4114	0.0016	0.0173	0.1434
MT-ND6	0.0367	0.0036	0.1711	0.0960	0.0714	0.4315	0.0054	0.0060	0.0810	0.0005	0.0484	0.0112	0.4724	0.0266	0.0400	0.0248

Supplementary data 3. Spearman correlation p-values. Analysis of bulk transcriptomic data of muscle biopsies from 44 DM patients tested positive for myositis-specific autoantibodies against NXP2 (n = 14), TIF1γ (n = 12), Mi2 (n = 12) and MDA5 (n = 6), and from 33 histologically normal muscle biopsies focusing on mitochondrial genes. *n* corresponds to independent subjects. Correlation of the expression of these mitochondrial genes with IFNγ-induced genes and other genes related to myositis disease activity: immune (*CD3E*, *CD4*, *CD8A*, *CD14*, *CD68*, *NCAM1*), myofiber regenerative (*MYOG*, *PAX7*, *MYH3*, *MYH8*) and mature myofiber markers (*ACTA1*, *MYH1*, *MYH2*). Source data are provided as a Source Data file.

Supplementary Table 4. glucose levels in the serum of *Icos*$^{-/-}$ NOD mice (Mean ± SD)

	**Glucose levels (mg/dl)**	**Hyperglycemia (cutoff 200mg/dl)**
Icos$^{-/-}$ NOD (Exp pNAC)	87.89 ± 22.83	No (0/16)
Icos$^{-/-}$ NOD + pNAC	129.1 ± 92.69	No (1/19)
Icos$^{-/-}$ NOD (Exp cNAC)	74.03 ± 50.26	No (0/16)
Icos$^{-/-}$ NOD + cNAC	94.83 ± 35.83	No (0/20)
Icos$^{-/-}$ NOD (Exp anti-IFNγ)	103.26 ± 50.82	No (0/4)
Icos$^{-/-}$ NOD + anti-IFNγ	100.3 ± 20.88	No (0/6)
Diabetic mice (Control +)	714.45 ± 207.43	Yes (6/6)

Supplementary Table 4. glucose levels in the serum of *Icos*$^{-/-}$ NOD mice. Glucose was measured in the serum of mice with a colorimetric commercial kit (Sigma). Data represents Mean ± SD. Serum from two independent experiments were included. For the preventive NAC experiments (pNAC), n=16 *Icos*$^{-/-}$ NOD, n=19 *Icos*$^{-/-}$ NOD + pNAC. For the curative NAC experiments (cNAC), n=16 *Icos*$^{-/-}$ NOD, n=20 *Icos*$^{-/-}$ NOD + cNAC. For the anti-IFNγ experiments, n=4 *Icos*$^{-/-}$ NOD, n=6 *Icos*$^{-/-}$ NOD + anti-IFNγ. A positive control for hyperglycemia consisted on alloxan-induced diabetic *Icos*$^{+/+}$ NOD mice (n=6). Samples over 200mg/dl were considered as hyperglycemic. For all, *n* corresponds to independent mice. Source data are provided as a Source Data file.

Supplementary Table 5. Sequences of the primers used for qRT-PCR analyses

Symbol	Forward	Reverse
Apc	GGAGTGGCAGAAAGCAACAC	CCACACGTGTAGCTGGACTC
Bax	GTGAGCGGCTGCTTGTCT	GGTCCCGAAGTAGGAGAGGA
Casp3	GAGGCTGACTTCCTGTATGCTT	AACCACGACCCGTCCTTT
Casp9	TGCAGTCCCTCCTTCTCAG	GCTTTTTCCGGAGGAAGTTAAA
Cat	GGAGGCGGGAACCCAATA	CAAAGTGTGCCATCTCGTCAGT
Ccl2	AAAAACCTGGATCGGAACCAA	CGGGTCAACTTCACATTCAAAG
Ccs	TCAAGGGTATGGGCAGTAGC	CACAGCCCTCCAGAATGG
Cxcl9	TCTGCCATGAAGTCCGCTG	5CAGGAGCATCGTGCATTCCT
Cxcl10	TGCTGGGTCTGAGTGGGACT	CCCTATGGCCCTCATTCTCAC
Ehd2	GAGACTTTCCTGACTGGAGA	TAGCGAGTGGAACTTGGTTGA
Fth1	ACGCAAGATGGGTGCCCCTGA	ACCGTGTCCCAGGGTGTGCTT
Gbp2	CTGCACTATGTGACGGAGCTA	GAGTCCACACAAAGGTTGGAAA
Gpx3	GGCTTCCCTTCCAACCAATT	CACCTGGTCGAACATACTTGAGACT
Gpx4	GGGCCGTCTGAGCCGCTTAC	TCGCGGGATGCACACATGGT
Gsr	GCCTTTACCCCGATGTATCA	AATGCCAACCACCTTTTCCT
Gss	CAGCTGTGCACCGACACGTTCT	GGCCAGTCCCTTGCTGGGGT
Hsp90ab1	CAGAAATTGCCCAGCTCATGT	CCGTCAGGCTCTCATATCGAA
Ifnb	AAGAGTTACACTGCCTTTGCC	CACTGTCTGCTGGTGGAGTTC
Ifng	TGCTGATGGGAGGAGATGTCT	TTTCTTTCAGGGACAGCCTGTT
Jun	CCAGAAGATGGTGTGGTGTTT	CTGACCCTCTCCCCTTGC
Map2k1	GCGCGCTCCCTGCTGAGTTG	AGGCCTCCAGGTTGGTCTCGG
Mapk1	TTGGTCAGGACAAGGGCTCAGAGGA	GCTGAGACGGGCTGAAGACAGG
Nfkb1	TTTCGACTACGCAGTGACGG	GAGCGTGGAGGTGGATGATG
Nono	TTAGTGAGGACTGCGAGGCA	TTCTGCTTCTCCAAGTTAAAGGCT
Park7	TGGGTACACGTCGGGTGCGA	TTAAAGACTGCAGCCGCGCCTC
Ppp1r15b	TGCTGGAGAAAGATACACCCATA	AATTCTTCCCATGGTCCTTTG
Prdx1	GTGAGACCTGTGGCTCGAC	TGTCCATCTGGCATAACAGC
Prdx5	CGAGTCCTGGGCTGCAAA	CACACTCCCAACCTGCTTCTTT
Rplp0	TCCTATAAAAGGCACACGCGGGCA	AGACGATGTCACTCCAACGAGGACG
Sp1	GCTGCCACCATGAGCGACCAA	CACCGCCACCATTGCCGCTA
Srxn1	AGGGGCTTCTGCAAACCTA	TGGCATAGCTACCTCACTGCT
Tfrc	GAGGCGCTTCCTAGTACTCCCTTGT	GCCGAGCAAGGCTAAACCGGG
Txnrd1	ACCGTGGGCGTGAAGATAAA	GATGTCACCGATGGCGTAGAT
Txnrd3	GTGAACGTAGGCTGTATTCCAAAGA	TTGTGCTTCACCTGCTGGTTATA

Supplementary Table 6. Antibodies used in immunofluorescence

Antibodies used in spatial transcriptome analysis			
**Primary antibodies**	**Manufacturer**	**Catalog number**	**Dilution**
anti-CD45 (rabbit monoclonal, clone D3F8Q)	Cell Signal Technology	70257S	concentration 20.00 ug/mL
anti-desmin (rabbit monoclonal, clone Y66)	Abcam	ab185033	concentration 5.00 ug/mL
**Antibodies used in immunofluorescence (other than spatial transcriptome analysis)**			
**Primary antibodies**	**Manufacturer**	**Catalog number**	**Dilution**
anti-CD4 (rat monoclonal, clone RM4-5)	BD pharmingen	550280	1:400
anti-CD8a (rabbit polyclonal)	Invitrogen	PA581344	1:400
anti-CD45 (rat monoclonal, clone 30-F11)	Sony	1115560	1:300
anti-B220 (rat monoclonal, clone RA3-6B2)	Sony	1116180	1:400
anti-F4/80 (rat monoclonal, clone BM8)	ebioscience	14-4801-82	1:400
anti-laminin (rabbit polyclonal)	Dako	Z0097	1:400
**Secondary antibodies**	**Manufacturer**	**Catalog number**	**Dilution**
CY3 Donkey F(AB')2 anti-rat IGG (H+L)	Jackson Immunoresearch	712-166-153	1:400
CY5 Goat anti-rat IGG (H+L)	Invitrogen	A10525	1:400
AF 488 Chicken anti-rabbit IGG (H+L)	Invitrogen	A21441	1:400
