# Supplementary material for: IFNγ causes mitochondrial dysfunction and oxidative stress in myositis
Source: Nat Commun. 2024 Jun 26;15:5403. doi: 10.1038/s41467-024-49460-1 (PMC11208592; doi:10.1038/s41467-024-49460-1)
Supplement: Supplementary file 2 — Reporting Summary [file 41467_2024_49460_MOESM2_ESM.pdf]

nature portfolio

Corresponding author(s): Olivier Boyer

Last updated by author(s): 17/05/2024

Reporting Summary

Nature Portfolio wishes to improve the reproducibility of the work that we publish. This form provides structure for consistency and transparency in reporting. For further information on Nature Portfolio policies, see our Editorial Policies and the Editorial Policy Checklist.

Statistics

For all statistical analyses, confirm that the following items are present in the figure legend, table legend, main text, or Methods section.

n/a	Confirmed	
☐	☒	The exact sample size (*n*) for each experimental group/condition, given as a discrete number and unit of measurement
☐	☒	A statement on whether measurements were taken from distinct samples or whether the same sample was measured repeatedly
☐	☒	The statistical test(s) used AND whether they are one- or two-sided<br>*Only common tests should be described solely by name; describe more complex techniques in the Methods section.*
☒	☐	A description of all covariates tested
☐	☒	A description of any assumptions or corrections, such as tests of normality and adjustment for multiple comparisons
☐	☒	A full description of the statistical parameters including central tendency (e.g. means) or other basic estimates (e.g. regression coefficient) AND variation (e.g. standard deviation) or associated estimates of uncertainty (e.g. confidence intervals)
☐	☒	For null hypothesis testing, the test statistic (e.g. *F*, *t*, *r*) with confidence intervals, effect sizes, degrees of freedom and *P* value noted<br>*Give P values as exact values whenever suitable.*
☒	☐	For Bayesian analysis, information on the choice of priors and Markov chain Monte Carlo settings
☒	☐	For hierarchical and complex designs, identification of the appropriate level for tests and full reporting of outcomes
☒	☐	Estimates of effect sizes (e.g. Cohen's *d*, Pearson's *r*), indicating how they were calculated

Our web collection on statistics for biologists contains articles on many of the points above.

Software and code

Policy information about availability of computer code

Data collection

Proteomics data was collected with a Q-exactive Plus Orbitrap™ Mass Spectrometer equipped with a nanoESI source and coupled to a chromatographic system, RSLCnano Ultimate 3000.
RNAs for spatial transcriptome analysis were collected with a GeoMX DSP device.
Spatial transcriptome data was generated with a Illumina NovaSeq6000 device.
Human transcriptome data was generated with a Illumina HiSeq 4000 device.
Tissue images were obtained with a Zeiss Axioscope 7 microscope (brightfield) equipped with Zen software and a Leica Thunder Tissue Imager system (fluorescence) equipped with LAS Software (Leica Application Suite 3.7.6.25997).
Electron microscopy images were obtained with a Tecnai 12 electron microscope (muscle tissue images) or a Morgagni 268D electron microscope (myotube in vitro images).
qPCR data was collected with a ABI Prism 7500 Sequence Detection System (oxidative stress panel) or a LightCycler 480 System SW 1.5.1 (chemokine/cytokine analysis).
Electron paramagnetic resonance was performed with a MiniScope MS-200 (Magnettech).
Mitochondrial function was assessed using high-resolution oxygraphy device (O2K, Oroboros instruments).
Catwalk data collection was perfomed with a Catwalk device from Noldus Information Technology.
Grip strength data collection was perfomed with a Bioseb Grip test device v3.40.
Muscle strength after electric stimulation was obtained with an isometric transducer (PowerLab Station, AD Instruments) and LabChart v7 software (AD instruments).
Addressable laser bead immunoassay (ALBIA) was performed with a Bio-Plex™ apparatus using the Bio-Plex™ Manager software v4.0 (Bio-Rad).

Data analysis	Proteomics data were analysed with Progenesis LC-MS software (Non-Linear Dynamics, v4.1), Ingenuity Pathway Analysis (IPA 2.4 (2019Q2) and STRING v12.0 (https://string-db.org/).<br>Spatial transcriptome data analysis was performed with Nanostring GeoMx Suite software v3.1.0.218.<br>For human transcriptomic analysis, reads were demultiplexed using bcl2fastq/2.20.0 and preprocessed using fastp/0.21.0. The abundance of each gene was generated using Salmon/1.5.2 and quality control output was summarized using multiqc/1.11. Counts were normalized using the Trimmed Means of M values (TMM) from edgeR/3.34.1 for graphical analysis. Differential expression was performed using limma/3.48.3.<br>Tissue image analysis was performed using Fiji software (ImageJ v2.3.0/1.53f51, Java 1.8.0_265).<br>Electron paramagnetic resonance data was analyzed with ANALYSIS v.2.02 software from Magnettech.<br>Catwalk data analysis was performed usng Catwalk XT v10.5.<br>Muscle strength after electric stimulation was analysed with LabChart v7 software.<br>Statistical analysis was performed using Graphpad Prism v8.4.3.

For manuscripts utilizing custom algorithms or software that are central to the research but not yet described in published literature, software must be made available to editors and reviewers. We strongly encourage code deposition in a community repository (e.g. GitHub). See the Nature Portfolio guidelines for submitting code & software for further information.

Data

Policy information about availability of data

All manuscripts must include a data availability statement. This statement should provide the following information, where applicable:

- Accession codes, unique identifiers, or web links for publicly available datasets
- A description of any restrictions on data availability
- For clinical datasets or third party data, please ensure that the statement adheres to our policy

Human transcriptome data were deposited into the Gene Expression Omnibus database under accession number GSE220915 and are available at the following URL: https://www.ncbi.nlm.nih.gov/geo/query/acc.cgi?acc=GSE220915. The mass spectrometry proteomics data have been deposited to the ProteomeXchange Consortium via the PRIDE partner repository with the dataset identifier PXD048004. Nanostring spatial transcriptome data were deposited into the Gene Expression Omnibus database under accession number GSE262352 and are available at the following URL: https://www.ncbi.nlm.nih.gov/geo/query/acc.cgi?acc=GSE262352. Source data are provided with this paper.

Research involving human participants, their data, or biological material

Policy information about studies with human participants or human data. See also policy information about sex, gender (identity/presentation), and sexual orientation and race, ethnicity and racism.

Reporting on sex and gender	The data was collected form a publicly available repository (https://www.ncbi.nlm.nih.gov/geo/query/acc.cgi?acc=GSE220915). Information about the composition of the study participants is available in the source manuscript from the GEO source. Genetic sex for human participants was self-reported and verified by the transcriptomic profile using the expression of Y-chromosome genes TTTY14, DDX3Y, and PRKY.
Reporting on race, ethnicity, or other socially relevant groupings	The data was collected form a publicly available repository (https://www.ncbi.nlm.nih.gov/geo/query/acc.cgi?acc=GSE220915). Patient and histologically normal samples were obtained irrespective of race, ethnicity or other socially relevant groupings.
Population characteristics	The data was collected form a publicly available repository (https://www.ncbi.nlm.nih.gov/geo/query/acc.cgi?acc=GSE220915). Information about the composition of the study participants is available in the source manuscript from the GEO source. Patients met the ACR criteria for DM74 and tested positive for one of the following myositis-specific autoantibodies (MSA): anti-NXP2, anti-Mi2, anti-TIF1g or anti-MDA5. ELISA, immunoprecipitation of proteins produced by in vitro transcription and translation (IVTT-IP), line blotting (EUROLINE myositis profile), and immunoprecipitation from 35S-methionine-labeled HeLa cell lysates were used to test for autoantibodies. Histologically normal muscle biopsies were obtained from the National Institutes of Health (n = 13), the University of Kentucky Skeletal Muscle Biobank (n = 8), and the Johns Hopkins Neuromuscular Pathology Laboratory (n = 12).
Recruitment	The data was collected form a publicly available repository (https://www.ncbi.nlm.nih.gov/geo/query/acc.cgi?acc=GSE220915). All muscle biopsies were obtained from individuals enrolled in institutional review board-approved (IRB) longitudinal cohorts from the National Institutes of Health in Bethesda, MD; the Johns Hopkins Myositis Center in Baltimore, MD; the Vall d'Hebron Hospital, and the Clinic Hospital in Barcelona.
Ethics oversight	This study was approved by the NIH, the Johns Hopkins, the Clinic, and the Vall d'Hebron Hospitals IRBs. Each participant signed a written informed consent.

Note that full information on the approval of the study protocol must also be provided in the manuscript.

Field-specific reporting

Please select the one below that is the best fit for your research. If you are not sure, read the appropriate sections before making your selection.

☒ Life sciences ☐ Behavioural & social sciences ☐ Ecological, evolutionary & environmental sciences

For a reference copy of the document with all sections, see nature.com/documents/nr-reporting-summary-flat.pdf

Life sciences study design

All studies must disclose on these points even when the disclosure is negative.

Sample size	No prior sample size calculation was performed. Sample sizes in the experiments were based on our previous experience with this disease model (Briet et al, Front Immunol. 2017;8:287. PMID: 28424681; Bourdenet et al, Neuropathol Appl Neurobiol. 2023;49(1):e12889. PMID: 36751013) and on availability of mice. Numbers are included in the manuscript text (Figure legends and/or material and methods).
Data exclusions	Any outliers were excluded by using the Dixon's Q test (Q95%). These are noted in the source data Table.
Replication	All experiments were successfully repeated in duplicate except for:<br>- proteome analysis, which included 5 mice per group<br>- Nanostring spatial transcriptomics, which included 4 Icos+/+ NOD mice (4 AOI) and 6 Icos-/- NOD mice (14AOI)<br>- human transcriptome analysis, which included 44 DM patients and 33 histologically normal controls<br>- in vitro studies, were two wells/treatment replicates were analysed
Randomization	Mice were used according to genotype and matched for age. Mice were tagged and selected randomly before treatment assignment in the preventive experimental setting. For the curative experimental setting, mice reaching a score of 2 were randomly assigned to no-treatment/treatment groups until reaching a minimum of n=8 mice/group. For in vitro experiments, individual wells were randomly assigned to different experimental conditions. No additional randomization was used during other data collection.
Blinding	Investigators performing data collection and analysis were blinded to the mouse genotype/treatment, with the exception of transcriptome analysis, where experimental method prevented blinding.

Behavioural & social sciences study design

All studies must disclose on these points even when the disclosure is negative.

Study description	*Briefly describe the study type including whether data are quantitative, qualitative, or mixed-methods (e.g. qualitative cross-sectional, quantitative experimental, mixed-methods case study).*
Research sample	*State the research sample (e.g. Harvard university undergraduates, villagers in rural India) and provide relevant demographic information (e.g. age, sex) and indicate whether the sample is representative. Provide a rationale for the study sample chosen. For studies involving existing datasets, please describe the dataset and source.*
Sampling strategy	*Describe the sampling procedure (e.g. random, snowball, stratified, convenience). Describe the statistical methods that were used to predetermine sample size OR if no sample-size calculation was performed, describe how sample sizes were chosen and provide a rationale for why these sample sizes are sufficient. For qualitative data, please indicate whether data saturation was considered, and what criteria were used to decide that no further sampling was needed.*
Data collection	*Provide details about the data collection procedure, including the instruments or devices used to record the data (e.g. pen and paper, computer, eye tracker, video or audio equipment) whether anyone was present besides the participant(s) and the researcher, and whether the researcher was blind to experimental condition and/or the study hypothesis during data collection.*
Timing	*Indicate the start and stop dates of data collection. If there is a gap between collection periods, state the dates for each sample cohort.*
Data exclusions	*If no data were excluded from the analyses, state so OR if data were excluded, provide the exact number of exclusions and the rationale behind them, indicating whether exclusion criteria were pre-established.*
Non-participation	*State how many participants dropped out/declined participation and the reason(s) given OR provide response rate OR state that no participants dropped out/declined participation.*
Randomization	*If participants were not allocated into experimental groups, state so OR describe how participants were allocated to groups, and if allocation was not random, describe how covariates were controlled.*

Ecological, evolutionary & environmental sciences study design

All studies must disclose on these points even when the disclosure is negative.

Study description	*Briefly describe the study. For quantitative data include treatment factors and interactions, design structure (e.g. factorial, nested, hierarchical), nature and number of experimental units and replicates.*
Research sample	*Describe the research sample (e.g. a group of tagged Passer domesticus, all Stenocereus thurberi within Organ Pipe Cactus National Monument), and provide a rationale for the sample choice. When relevant, describe the organism taxa, source, sex, age range and any manipulations. State what population the sample is meant to represent when applicable. For studies involving existing datasets, describe the data and its source.*

Sampling strategy	*Note the sampling procedure. Describe the statistical methods that were used to predetermine sample size OR if no sample-size calculation was performed, describe how sample sizes were chosen and provide a rationale for why these sample sizes are sufficient.*
Data collection	*Describe the data collection procedure, including who recorded the data and how.*
Timing and spatial scale	*Indicate the start and stop dates of data collection, noting the frequency and periodicity of sampling and providing a rationale for these choices. If there is a gap between collection periods, state the dates for each sample cohort. Specify the spatial scale from which the data are taken*
Data exclusions	*If no data were excluded from the analyses, state so OR if data were excluded, describe the exclusions and the rationale behind them, indicating whether exclusion criteria were pre-established.*
Reproducibility	*Describe the measures taken to verify the reproducibility of experimental findings. For each experiment, note whether any attempts to repeat the experiment failed OR state that all attempts to repeat the experiment were successful.*
Randomization	*Describe how samples/organisms/participants were allocated into groups. If allocation was not random, describe how covariates were controlled. If this is not relevant to your study, explain why.*
Blinding	*Describe the extent of blinding used during data acquisition and analysis. If blinding was not possible, describe why OR explain why blinding was not relevant to your study.*

Did the study involve field work? ☐ Yes ☐ No

Field work, collection and transport

Field conditions	*Describe the study conditions for field work, providing relevant parameters (e.g. temperature, rainfall).*
Location	*State the location of the sampling or experiment, providing relevant parameters (e.g. latitude and longitude, elevation, water depth).*
Access & import/export	*Describe the efforts you have made to access habitats and to collect and import/export your samples in a responsible manner and in compliance with local, national and international laws, noting any permits that were obtained (give the name of the issuing authority, the date of issue, and any identifying information).*
Disturbance	*Describe any disturbance caused by the study and how it was minimized.*

Reporting for specific materials, systems and methods

We require information from authors about some types of materials, experimental systems and methods used in many studies. Here, indicate whether each material, system or method listed is relevant to your study. If you are not sure if a list item applies to your research, read the appropriate section before selecting a response.

Materials & experimental systems

n/a	Involved in the study	
☐	☒	Antibodies
☐	☒	Eukaryotic cell lines
☒	☐	Palaeontology and archaeology
☐	☒	Animals and other organisms
☒	☐	Clinical data
☒	☐	Dual use research of concern
☒	☐	Plants

Methods

n/a	Involved in the study	
☒	☐	ChIP-seq
☒	☐	Flow cytometry
☒	☐	MRI-based neuroimaging

Antibodies

Antibodies used

Primary antibodies (Nanostring)
• anti-CD45 (Cell Signal Technology, cat. number 70257S, lot number 81143S, rabbit monoclonal, clone D3F8Q, concentration 20.00 ug/mL)
• anti-desmin (Abcam, cat. number ab185033, lot number GR3269405-8, rabbit monoclonal, clone Y66, concentration 5.00 ug/mL)

Primary antibodies (other than Nanostring)
• anti-CD4 (BD pharmingen, cat. number 550280, lot number 8085954, rat monoclonal, clone RM4-5, dilution 1:400)
• anti-CD8a (Invitrogen, cat. number PA581344, lot number Y4051343, rabbit polyclonal, dilution 1:400)
• anti-CD45 (Sony, cat. number 1115560, lot number 214709, rat monoclonal, clone 30-F11, dilution 1:300)
• anti-B220 (Sony, cat. number 1116180, lot number 109663, rat monoclonal, clone RA3-6B2, dilution, 1:400)
• anti-F4/80 (ebioscience, cat. number 14-4801-82, lot number E04272-1635, rat monoclonal, clone BM8, dilution 1:400)

• anti-laminin (Dako, cat. number Z0097, lot number 20025736, rabbit polyclonal, dilution 1:400)

Secondary antibodies
• CY3 Donkey F(AB')2 anti-rat IGG (H+L) (Jackson Immunoresearch, cat. number 712-166-153, lot number 135846, dilution 1:400)
• CY5 Goat anti-rat IGG (H+L) (Invitrogen, Cat. number A10525, lot number 2387442, dilution 1:400)
• AF 488 Chicken anti-rabbit IGG (H+L) (Invitrogen, Cat. number A21441, lot number 2387456, dilution 1:400)

Validation

All antibodies used in the study are commercially available and validated by the manufacturers as indicated in the website and corresponding datasheet. The concentrations of antibodies used in the study were confirmed on the basis of concentration test experiments.

• Anti-CD4 (clone RM4-5) was validated as reported in https://www.bdbiosciences.com/en-us/products/reagents/flow-cytometry-reagents/research-reagents/single-color-antibodies-ruo/purified-rat-anti-mouse-cd4.550280 and was used in our previous publication: Bourdenet et al, Neuropathol Appl Neurobiol. 2023;49(1):e12889. PMID: 36751013.
• Anti-desmin (clone Y66) was validated as reported in https://www.abcam.com/en-fr/products/primary-antibodies/alexa-fluor-488-desmin-antibody-y66-cytoskeleton-marker-ab185033.
• Anti-CD8a was validated as reported in https://www.thermofisher.com/antibody/product/CD8-alpha-Antibody-Polyclonal/PA5-81344
• Anti-CD45 antibody (clone 30-F11), was validated as reported in https://www.sonybiotechnology.com/us/apc-anti-mouse-cd45-14 and was used in Mei et al, Front Immunol. 2021 15;12:617163. PMID: 33659003.
• Anti-CD45 antibody (clone D3F8Q), was validated as reported in https://www.cellsignal.com/products/primary-antibodies/cd45-d3f8q-rabbit-mab/70257
• Anti-B220 (clone RA3-6B2) was validated as reported in https://www.sonybiotechnology.com/us/percp-cy5-5-anti-mouse-human-cd45r-b220-6 and was used in our previous publication: Bourdenet et al, Neuropathol Appl Neurobiol. 2023;49(1):e12889. PMID: 36751013.
• Anti-F4/80 antibody (clone BM8) was validated as reported in https://www.thermofisher.com/antibody/product/F4-80-Antibody-clone-BM8-Monoclonal/14-4801-82 and was used in our previous publication: Briet et al, Front Immunol. 2017;8:287. PMID: 28424681.
• Anti-anti-laminin has been extensively used in the literature including our previous publications: Bourdenet et al, Neuropathol Appl Neurobiol. 2023;49(1):e12889. PMID: 36751013; Julien et al, Biomedicines. 2022;10(8):2036. PMID: 36009583.

Eukaryotic cell lines

Policy information about cell lines and Sex and Gender in Research

Cell line source(s)

human myoblast cell line LHCN-M2 (Evercyte, Cat#: CkHT-040-231-2).

Authentication

LHCN-M2 is a commercially available cell line. It was developed from human satellite cells (from the pectoralis major muscle) by transduction with retroviral vectors containing the cdk-4 and hTERT gene. The cell line was continuously cultured for more than 200 population doublings without showing signs of growth retardation or replicative senescence and shows the typical myoblast morphology (Zhu, Ch.-H. et al. 2007, Aging Cell, 6(4):515-23, [PubMed UID: 17559502])

Mycoplasma contamination

The cell line LHCN-M2 was cultured at the Cell Culture Facility of the IGBMC institute. LHCN-M2 cells tested negative for mycoplasma upon aliquot freezing. No contamination was detected in routine laboratory tests.

Commonly misidentified lines
(See ICLAC register)

No commonly misidentified lines were used in this study.

Palaeontology and Archaeology

Specimen provenance

Provide provenance information for specimens and describe permits that were obtained for the work (including the name of the issuing authority, the date of issue, and any identifying information). Permits should encompass collection and, where applicable, export.

Specimen deposition

Indicate where the specimens have been deposited to permit free access by other researchers.

Dating methods

If new dates are provided, describe how they were obtained (e.g. collection, storage, sample pretreatment and measurement), where they were obtained (i.e. lab name), the calibration program and the protocol for quality assurance OR state that no new dates are provided.

☐ Tick this box to confirm that the raw and calibrated dates are available in the paper or in Supplementary Information.

Ethics oversight

Identify the organization(s) that approved or provided guidance on the study protocol, OR state that no ethical approval or guidance was required and explain why not.

Note that full information on the approval of the study protocol must also be provided in the manuscript.

Animals and other research organisms

Policy information about studies involving animals; ARRIVE guidelines recommended for reporting animal research, and Sex and Gender in Research

Laboratory animals	Icos-/- and Icos+/+ mice on a NOD background (Prevot et al, Eur.J.Immunol. 40:2267-2276) ranging from 8-35 weeks of age were used in our studies. Mice were housed under a 12 h light/dark cycle with food pellets and drinking water provided ad libitum. Room temperature was maintained within the range of 21-23°C and relative humidity ranged between 45-55%.
Wild animals	No wild animals were used in this study.
Reporting on sex	We chose to use only females in this model where the disease is very slowly progressive disease, because the incidence of spontaneous myopathy is significantly higher in females (70%) than in males (20%) as we reported previously (Prevot, N. et al Eur J Immunol. 2010 Aug;40(8):2267-76. doi: 10.1002/eji.201040416).
Field-collected samples	No field-collected samples were used for this study.
Ethics oversight	All protocols were performed with the agreement of the local animal ethics committee ("Comité National de Réflexion Ethique sur l'EXpérimentation Animale") and approved by the committee for animal experimentation from the French Ministry of High Education and Research ("Ministère de l'Enseignement Superieur et de la Recherche", authorisation number APAFIS#8780).

Note that full information on the approval of the study protocol must also be provided in the manuscript.

Clinical data

Policy information about clinical studies

All manuscripts should comply with the ICMJE guidelines for publication of clinical research and a completed CONSORT checklist must be included with all submissions.

Clinical trial registration	*Provide the trial registration number from ClinicalTrials.gov or an equivalent agency.*
Study protocol	*Note where the full trial protocol can be accessed OR if not available, explain why.*
Data collection	*Describe the settings and locales of data collection, noting the time periods of recruitment and data collection.*
Outcomes	*Describe how you pre-defined primary and secondary outcome measures and how you assessed these measures.*

Dual use research of concern

Policy information about dual use research of concern

Hazards

Could the accidental, deliberate or reckless misuse of agents or technologies generated in the work, or the application of information presented in the manuscript, pose a threat to:

No	Yes	
☒	☐	Public health
☒	☐	National security
☒	☐	Crops and/or livestock
☒	☐	Ecosystems
☒	☐	Any other significant area

Experiments of concern

Does the work involve any of these experiments of concern:

No	Yes	
☒	☐	Demonstrate how to render a vaccine ineffective
☒	☐	Confer resistance to therapeutically useful antibiotics or antiviral agents
☒	☐	Enhance the virulence of a pathogen or render a nonpathogen virulent
☒	☐	Increase transmissibility of a pathogen
☒	☐	Alter the host range of a pathogen
☒	☐	Enable evasion of diagnostic/detection modalities
☒	☐	Enable the weaponization of a biological agent or toxin
☒	☐	Any other potentially harmful combination of experiments and agents

Plants

Seed stocks: *Report on the source of all seed stocks or other plant material used. If applicable, state the seed stock centre and catalogue number. If plant specimens were collected from the field, describe the collection location, date and sampling procedures.*

Novel plant genotypes: *Describe the methods by which all novel plant genotypes were produced. This includes those generated by transgenic approaches, gene editing, chemical/radiation-based mutagenesis and hybridization. For transgenic lines, describe the transformation method, the number of independent lines analyzed and the generation upon which experiments were performed. For gene-edited lines, describe the editor used, the endogenous sequence targeted for editing, the targeting guide RNA sequence (if applicable) and how the editor was applied.*

Authentication: *Describe any authentication procedures for each seed stock used or novel genotype generated. Describe any experiments used to assess the effect of a mutation and, where applicable, how potential secondary effects (e.g. second site T-DNA insertions, mosiacism, off-target gene editing) were examined.*

Flow Cytometry

Plots

Confirm that:

☐ The axis labels state the marker and fluorochrome used (e.g. CD4-FITC).

☐ The axis scales are clearly visible. Include numbers along axes only for bottom left plot of group (a 'group' is an analysis of identical markers).

☐ All plots are contour plots with outliers or pseudocolor plots.

☐ A numerical value for number of cells or percentage (with statistics) is provided.

Methodology

Sample preparation: *Describe the sample preparation, detailing the biological source of the cells and any tissue processing steps used.*

Instrument: *Identify the instrument used for data collection, specifying make and model number.*

Software: *Describe the software used to collect and analyze the flow cytometry data. For custom code that has been deposited into a community repository, provide accession details.*

Cell population abundance: *Describe the abundance of the relevant cell populations within post-sort fractions, providing details on the purity of the samples and how it was determined.*

Gating strategy: *Describe the gating strategy used for all relevant experiments, specifying the preliminary FSC/SSC gates of the starting cell population, indicating where boundaries between "positive" and "negative" staining cell populations are defined.*

☐ Tick this box to confirm that a figure exemplifying the gating strategy is provided in the Supplementary Information.

Magnetic resonance imaging

Experimental design

Design type: *Indicate task or resting state; event-related or block design.*

Design specifications	*Specify the number of blocks, trials or experimental units per session and/or subject, and specify the length of each trial or block (if trials are blocked) and interval between trials.*
Behavioral performance measures	*State number and/or type of variables recorded (e.g. correct button press, response time) and what statistics were used to establish that the subjects were performing the task as expected (e.g. mean, range, and/or standard deviation across subjects).*

Acquisition

Imaging type(s)	*Specify: functional, structural, diffusion, perfusion.*
Field strength	*Specify in Tesla*
Sequence & imaging parameters	*Specify the pulse sequence type (gradient echo, spin echo, etc.), imaging type (EPI, spiral, etc.), field of view, matrix size, slice thickness, orientation and TE/TR/flip angle.*
Area of acquisition	*State whether a whole brain scan was used OR define the area of acquisition, describing how the region was determined.*

Diffusion MRI ☐ Used ☐ Not used

Preprocessing

Preprocessing software	*Provide detail on software version and revision number and on specific parameters (model/functions, brain extraction, segmentation, smoothing kernel size, etc.).*
Normalization	*If data were normalized/standardized, describe the approach(es): specify linear or non-linear and define image types used for transformation OR indicate that data were not normalized and explain rationale for lack of normalization.*
Normalization template	*Describe the template used for normalization/transformation, specifying subject space or group standardized space (e.g. original Talairach, MNI305, ICBM152) OR indicate that the data were not normalized.*
Noise and artifact removal	*Describe your procedure(s) for artifact and structured noise removal, specifying motion parameters, tissue signals and physiological signals (heart rate, respiration).*
Volume censoring	*Define your software and/or method and criteria for volume censoring, and state the extent of such censoring.*

Statistical modeling & inference

Model type and settings	*Specify type (mass univariate, multivariate, RSA, predictive, etc.) and describe essential details of the model at the first and second levels (e.g. fixed, random or mixed effects; drift or auto-correlation).*
Effect(s) tested	*Define precise effect in terms of the task or stimulus conditions instead of psychological concepts and indicate whether ANOVA or factorial designs were used.*

Specify type of analysis: ☐ Whole brain ☐ ROI-based ☐ Both

Statistic type for inference<br>(See Eklund et al. 2016)	*Specify voxel-wise or cluster-wise and report all relevant parameters for cluster-wise methods.*
Correction	*Describe the type of correction and how it is obtained for multiple comparisons (e.g. FWE, FDR, permutation or Monte Carlo).*

Models & analysis

n/a	Involved in the study
☐	☐ Functional and/or effective connectivity
☐	☐ Graph analysis
☐	☐ Multivariate modeling or predictive analysis

Functional and/or effective connectivity	*Report the measures of dependence used and the model details (e.g. Pearson correlation, partial correlation, mutual information).*
Graph analysis	*Report the dependent variable and connectivity measure, specifying weighted graph or binarized graph, subject- or group-level, and the global and/or node summaries used (e.g. clustering coefficient, efficiency, etc.).*
Multivariate modeling and predictive analysis	*Specify independent variables, features extraction and dimension reduction, model, training and evaluation metrics.*
